# Association of glucose–lymphocyte ratio and short-term mortality in patients with sepsis complicated by ARDS during the acute phase: a multicenter retrospective cohort study

**DOI:** 10.3389/fcimb.2026.1771620

**Published:** 2026-03-19

**Authors:** Shucun Liu, Wenjing Du, Jiazhi Zhou, Xingxing Zhuang, Wei Xu, Chunyang Li, Jiaqiong Li, Hongjie Tang

**Affiliations:** 1 The Xuzhou Clinical College of Xuzhou Medical University, Xuzhou, Jiangsu, China; 2 Department of Critical Care Medicine, Xuzhou Central Hospital, Southeast University, Xuzhou, Jiangsu, China; 3 Department of General Surgery, Peixian Hospital of Traditional Chinese Medicine, Xuzhou, Jiangsu, China; 4 The Xuzhou Red Cross Blood Centre, Xuzhou, Jiangsu, China; 5 Department of Infectious Diseases, Affiliated Hospital of Xuzhou Medical University, Xuzhou, Jiangsu, China

**Keywords:** acute respiratory distress syndrome - ARDS, biomarker, glucose-to-lymphocyte ratio (GLR), mortality, sepsis

## Abstract

**Background:**

Sepsis may precipitate acute respiratory distress syndrome (ARDS), a life-threatening pulmonary complication characterized by high mortality. The glucose-to-lymphocyte ratio (GLR) is a novel composite biomarker has demonstrated prognostic significance across multiple diseases. However, its association with short-term mortality in patients with sepsis-induced ARDS has not yet been clearly established.

**Methods:**

In this multicenter retrospective cohort analysis, data from 2,485 individuals in the MIMIC-IV database were used to construct the primary derivation cohort, while an additional set of 298 patients from the Affiliated Hospital of Xuzhou Medical University served as an independent cohort for external validation. All-cause mortality at 28 days was defined as the primary outcome of the study. Least absolute shrinkage and selection operator (LASSO)–Boruta selected predictors. We evaluated the relationship between the GLR and mortality by employing multivariable Cox proportional hazards modeling, complemented by restricted cubic spline (RCS) and subgroup analyses. Receiver operating characteristic curves (ROC) and decision curve analysis (DCA) quantified incremental value of GLR over the Acute Physiology Score III (APS III) and the Simplified Acute Physiology Score II (SAPS II).

**Results:**

In the derivation cohort, higher GLR values were independently linked to an increased risk of 28-day mortality (adjusted HR 1.13 per unit increase, 95% CI: 1.053–1.211, P < 0.001). The finding was replicated in the external validation cohort. Short-term mortality increased linearly with rising GLR levels, as shown by RCS analysis. Subgroup analyses identified significant interactions: the prognostic value of GLR was significantly attenuated or lost in patients with high illness severity scores (SAPS II > 40, APS III > 53) or severe liver disease, while it remained robust in patients with lower severity scores and without severe liver disease. Incorporating GLR into APS III and SAPS II models improved their AUC values (APS III: 0.694 vs. 0.708; SAPS II: 0.678 vs. 0.696).

**Conclusions:**

An elevated GLR at admission independently predicts 28-day mortality in patients with sepsis-induced ARDS. This marker is especially useful for early risk stratification among patients without advanced liver dysfunction or severe physiological abnormalities.

## 1 Introduction

Sepsis arises when an infection triggers an aberrant host immune response, resulting in severe organ dysfunction and constituting one of the leading causes of global illness and death. Sepsis-induced acute respiratory distress syndrome (ARDS) accounts for more than one-third of all ARDS cases globally, with in-hospital mortality reaching 40% (Singer et al., 2016). ARDS represents a frequent and severe clinical condition, defined pathologically by widespread injury to the alveoli and manifested clinically as non-cardiogenic pulmonary edema accompanied by persistent, severe hypoxemia (Ma et al., 2025).

Sepsis and ARDS involve complex pathophysiological mechanisms characterized by profound immunometabolic imbalance and uncontrolled inflammation. Timely recognition of patients at elevated risk, together with prompt and appropriate risk classification, is crucial for informing treatment decisions and ultimately enhancing clinical outcomes. Clinically, various biomarkers such as C-reactive protein (CRP), procalcitonin (PCT), and comprehensive scoring systems (e.g., the SOFA score) are currently used to assess disease severity and prognosis. The prognostic value of composite inflammatory biomarkers has been demonstrated across a spectrum of acute conditions, from emergency general surgery to neurological events, underscoring their broad clinical relevance. However, the search for rapid, cost-effective composite indicators that can simultaneously reflect metabolic function and immunosuppression in specific high-risk populations remains a research hotspot (Vural et al., 2023; Vural et al., 2024; Lu et al., 2025). Blood glucose and lymphocyte counts reflect metabolic stress and immune status under the insult of sepsis; hyperglycemia in septic patients represents a stress response, while lymphocytes play a key role in immune function, and their reduction (lymphopenia) often indicates immune suppression during sepsis; therefore, the combined glucose-to-lymphocyte ratio (GLR) may serve as a more accurate and comprehensive prognostic indicator. GLR has been widely investigated for assessing disease severity and predicting mortality risk, and studies have shown that elevated GLR levels are associated with poor prognosis in conditions such as malignancies and cardiovascular events (Gan et al., 2025). Zhang et al. demonstrated that higher GLR values serve as an independent predictor of 28-day mortality among individuals with sepsis. Their analysis showed a nonlinear association and demonstrated that GLR provides superior prognostic performance compared with blood glucose or lymphocyte count alone (Leung et al., 2010). Previous investigations based on the MIMIC database have shown that elevated GLR levels function as an independent indicator of in-hospital mortality in patients with ARDS (Liu et al., 2021).

To date, no studies have specifically explored how the GLR relates to short-term mortality in individuals with sepsis-induced ARDS, a subgroup with particularly elevated risk. Accordingly, this investigation sought to determine whether GLR serves as an independent predictor of short-term mortality in this population by analyzing data from the MIMIC-IV database together with a multicenter cohort from the Affiliated Hospital of Xuzhou Medical University. The novelty of this study lies in further quantifying the incremental predictive value of incorporating GLR into existing critical illness scoring systems (APSIII, SAPSII) for mortality risk prediction, while conducting subgroup analyses to explore heterogeneity in prognostic performance of GLR.

## 2 Method

### 2.1 Study design and population

In this multicenter retrospective cohort analysis, we evaluated the association between the GLR and short-term mortality among patients presenting with sepsis complicated by ARDS. Data from the Medical Information Mart for Intensive Care IV(MIMIC-IV) database were employed to construct and analyze the derivation model, whereas an independent patient cohort from the Affiliated Hospital of Xuzhou Medical University was utilized for external validation to assess the stability and generalizability of the findings.

The primary study population was derived from version 3.1 of MIMIC-IV, a comprehensive, publicly accessible critical care database originating from a single center. It contains detailed clinical information on patients admitted to the intensive care units of Beth Israel Deaconess Medical Center (BIDMC) in the United States between 2008 and 2019. The study authors completed the required National Institutes of Health (NIH) training and passed the Collaborative Institutional Training Initiative (CITI Program) examination (certificate code: 12754211) to obtain database access. As all patient identifiers were anonymized, the Institutional Review Board (IRB) waived the need for informed consent.

The external validation cohort was drawn from the intensive care unit (ICU) of the Affiliated Hospital of Xuzhou Medical University. Clinical data were retrospectively collected from all patients who met the predefined inclusion and exclusion criteria between June 2024 and June 2025. Ethical approval for this investigation was granted by the Ethics Committee of the Affiliated Hospital of Xuzhou Medical University (approval number: XYFY2024-KL284-01). Owing to its retrospective design and reliance on anonymized data, ethical approval did not require individual informed consent.

Authorization to access and utilize the MIMIC-IV database was granted to the study’s principal investigators. This manuscript was prepared in accordance with the Strengthening the Reporting of Observational Studies in Epidemiology (STROBE) guidelines. This work followed the Strengthening the Reporting of Observational Studies in Epidemiology (STROBE)framework to ensure transparent and standardized reporting.

### 2.2 Population selection

**Figure 1:**
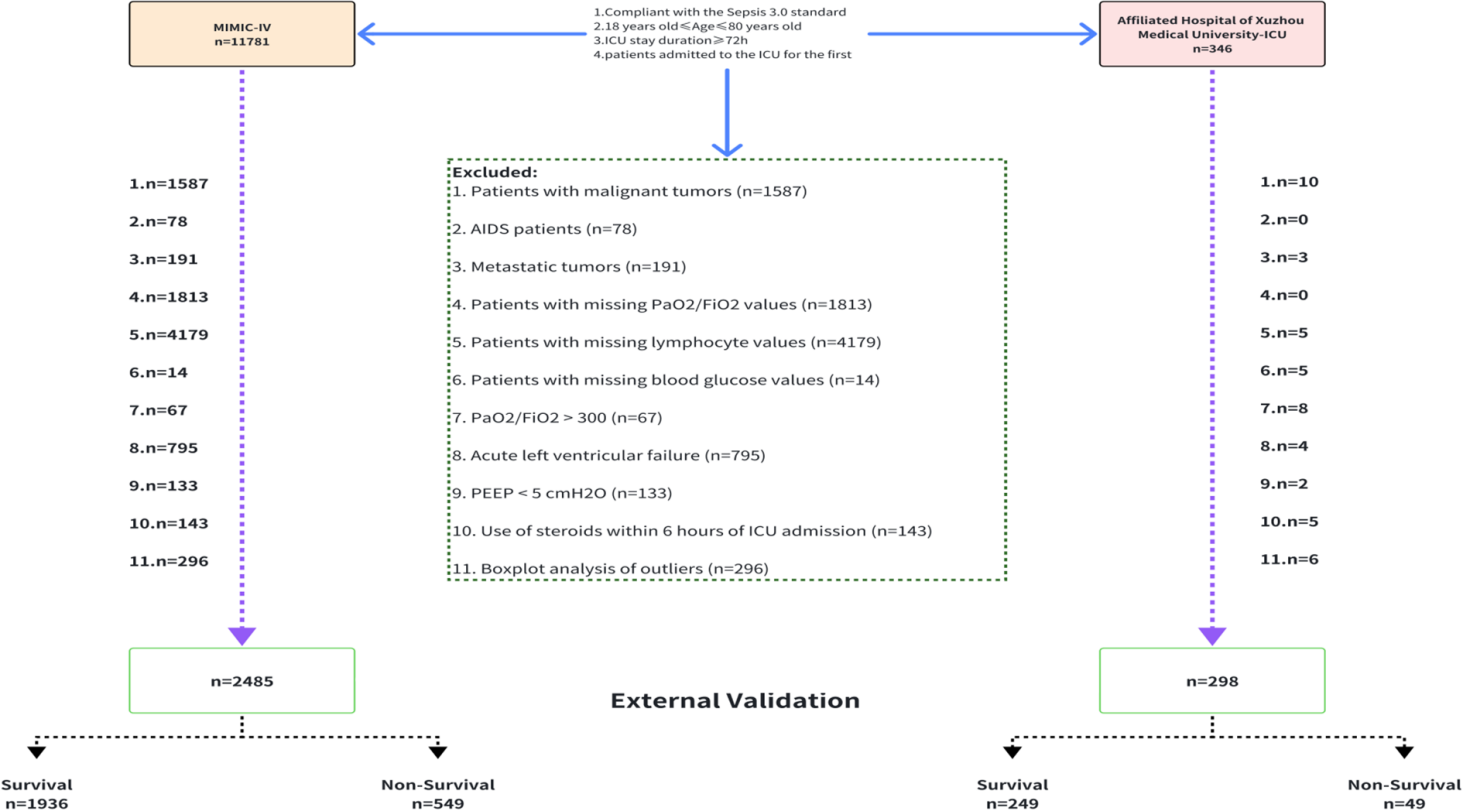
Flowchart of the study. ICU, Intensive care unit; AIDS, Acquired immunodeficiency syndrome; PaO2, Partial pressure of oxygen; FiO2, Fraction of inspiration O2; PEEP, Positive end-expiratory pressure.

Participants were eligible for inclusion provided that all of the following criteria were met: (1) first admission to the ICU and aged 18–80 years; (2) diagnosis of sepsis complicated by ARDS during the acute phase; and (3) ICU stay of at least 72 hours. The sepsis was defined according to the Third International Consensus Definitions (Sepsis-3.0) (Singer et al., 2016), and the diagnosis of ARDS was established according to the 2012 Berlin Definition (ARDS Definition Task Force et al., 2012). The acute phase of sepsis was defined as the period within 72 hours following a confirmed sepsis diagnosis. Exclusion criteria included: (1) active malignancy, including malignant tumors and metastatic cancer; (2) acquired immunodeficiency syndrome (AIDS); (3) receipt of glucocorticoid therapy within 6 hours prior to ICU admission; and (4) missing any of the following data at initial ICU assessment: blood glucose, lymphocyte count, arterial blood gas analysis (PaO_2_), or fraction of inspired oxygen (FiO_2_) (**Figure 1**).

### 2.3 Data extraction

Structured query language (SQL) was used to extract case information from the MIMIC-IV database (deployed on PostgreSQL 17.4), and data management and query operations were performed using Navicat Premium. The definitions and extraction procedures for key clinical variables—such as sepsis identification, calculation of organ function scores, and definitions of comorbidities were based on standardized scripts provided by the MIT Laboratory for Computational Physiology (LCP) in their official GitHub repository(https://github.com/MIT-LCP/mimic-iv)and were adapted according to the specific inclusion and exclusion criteria of this study.

The variables extracted in this study included the following: (1) demographic data: age, sex, height, weight, and BMI (kg/m²); (2) chronic comorbidities identified using ICD-9/10 codes: congestive heart failure, diabetes, chronic pulmonary disease, cerebrovascular disease, myocardial infarction, renal disease, and severe liver disease; (3) vital signs: temperature (T), heart rate (HR), respiratory rate (RR), systolic blood pressure (SBP), diastolic blood pressure (DBP), mean arterial pressure (MAP), and pulse oxygen saturation (SpO_2_); (4) laboratory indicators: white blood cell count (WBC), hemoglobin (Hb), hematocrit (HCT), red cell distribution width (RDW), lymphocytes, platelet count (PLT), C-reactive protein (CRP), creatinine (Cr), blood urea nitrogen (BUN), alanine aminotransferase (ALT), aspartate aminotransferase (AST), albumin (ALB), total bilirubin (TB), unconjugated bilirubin, lactate dehydrogenase (LDH), activated partial thromboplastin time (APTT), and blood glucose; (5) arterial blood gas parameters: pH, fraction of inspired oxygen (FiO_2_), arterial oxygen partial pressure (PaO_2_), arterial carbon dioxide partial pressure (PaCO_2_), blood lactate (Lac), calcium, anion gap, base excess, bicarbonate, sodium, globulin, and potassium; (6) organ support and complications: use of vasoactive agents (defined as the use of any vasopressor or inotropic drug), occurrence of acute kidney injury (AKI) based on KDIGO criteria, ventilatory parameters including positive end-expiratory pressure (PEEP) and tidal volume (VT); (7) critical illness scores: systemic inflammatory response syndrome (SIRS), sequential organ failure assessment (SOFA), acute physiology score III (APSIII), and simplified acute physiology score II (SAPSII), all calculated within 24 hours after ICU admission; (8) ARDS classification: patients were categorized as having mild, moderate, or severe ARDS according to PaO_2_/FiO_2_ levels, following “Acute respiratory distress syndrome: causes, pathophysiology, and phenotypes”: mild (200 mmHg < PaO_2_/FiO_2_ ≤ 300 mmHg), moderate (100 mmHg < PaO_2_/FiO_2_ ≤ 200 mmHg), and severe (PaO_2_/FiO_2_ ≤ 100 mmHg). Clinical laboratory results and severity evaluation scores were gathered within the first day of intensive care unit hospitalization. Multivariable analyses did not include variables with missing data greater than 20% to improve reliability (**Supplementary Table 1**).

### 2.4 Clinical outcomes

The primary endpoint of this study was 28-day all-cause mortality following ICU admission.

### 2.5 Calculation of GLR

The glucose-to-lymphocyte ratio (GLR) was calculated as follows: GLR = log_2_ [ICU admission blood glucose (mg/dL) ÷ (18 × lymphocyte count (mmol/L))]. Classification into survivor versus non-survivor groups was determined by 28-day ICU results. Furthermore, GLR values were divided into four quartiles (Q1–Q4), with Q1 serving as the reference category for comparisons across quartiles.

### 2.6 Statistical analysis

All statistical analyses were performed using R software (version 4.3.1) and Python (version 3.12). Results were deemed statistically significant if the two-sided P-value was under 0.05.

#### 2.6.1 Descriptive statistics and group comparisons

Continuous variables were first assessed for normality. Continuous variables exhibiting a normal distribution are expressed as mean ± standard deviation (x̄ ± s) and were compared using the independent-samples t-test. Continuous variables that do not follow a normal distribution are presented as median (interquartile range) [M (QR) or M (QL, QU)] and compared using the Mann-Whitney U test for two-group analyses or the Kruskal-Wallis test for comparisons involving multiple groups. For categorical variables, counts and percentages were reported, and comparisons were performed using Chi-square or Fisher’s exact tests according to data suitability.

#### 2.6.2 Handling of missing data

Missing data were handled using multiple imputation with chained equations (MICE package in R, 5 iterations). All variables included in the subsequent analyses were incorporated into the imputation model, and sensitivity analyses indicated that the distribution of the imputed data closely resembled that of the original dataset.(Kolmogorov-Smirnov test, P > 0.05).

#### 2.6.3 Survival analysis

Patients were divided into four quartiles (Q1–Q4) according to their GLR values. Kaplan-Meier survival analyses were performed to examine 28-day mortality, with differences across GLR quartiles evaluated using the log-rank test.

#### 2.6.4 Construction of multivariable prognostic models

A robust modeling approach was adopted involving the following steps:

##### 2.6.4.1 Variable selection

To prevent overfitting and identify the strongest predictors, two complementary machine learning methods were employed: LASSO regression (with the optimal λ determined by 10-fold cross-validation) and the Boruta algorithm (a random forest-based feature selection method). Variables were considered “important” if their importance score significantly exceeded that of shuffled “Shadow Max” features (p < 0.05).The final set of variables consisted of the intersection of results from both methods.

##### 2.6.4.2 Multicollinearity diagnosis

Prior to model construction, a Spearman correlation matrix was generated; variables with |r|≥0.7 were considered strongly correlated and excluded. In the final model, multicollinearity was evaluated using the Variance Inflation Factor (VIF), with a VIF greater than 5 considered indicative of significant collinearity and necessitating variable exclusion.

##### 2.6.4.3 Multivariable cox regression

Three sequential models were developed to assess the independent prognostic significance of GLR: Model 1 (unadjusted); Model 2 (adjusted for age and laboratory indices); and Model 3 (further adjusted for comorbidities and disease severity scores). GLR was analyzed both as a continuous variable (to calculate Hazard Ratios [HR] and 95% Confidence Intervals [CI]) and as an ordered categorical variable (quartiles) to test for linear trends (P for trend).

#### 2.6.5 Assessment of non-linear associations

Restricted cubic spline (RCS) analysis was performed to explore potential nonlinear associations between GLR and 28-day ICU mortality, with four knots placed at the 5th, 35th, 65th, and 95th percentiles.

#### 2.6.6 Subgroup analysis

To assess heterogeneity in the predictive efficacy of GLR, stratified analyses were conducted based on age, gender, diabetes, severe liver dysfunction, ARDS severity, SOFA score, APS III score, and SAPS II score. Interactions were evaluated by introducing interaction terms between GLR and subgroup variables into the Cox model.

#### 2.6.7 Model performance evaluation

The incremental value of GLR to existing scoring systems (APS III, SAPS II) was assessed by comparing the Area Under the Receiver Operating Characteristic (ROC) Curve (AUC). Differences in AUC were tested using the DeLong test with 1,000 bootstrap resamples. Decision Curve Analysis (DCA) was utilized to evaluate the clinical net benefit of the models.

#### 2.6.8 External validation

The final prognostic model’s generalizability and stability were assessed using an independent external cohort drawn from the ICU of the Affiliated Hospital of Xuzhou Medical University.

## 3 Results

### 3.1 Patient selection and baseline characteristics

Overall, 2,783 patients were included, comprising 2,485 individuals from the MIMIC-IV database and 298 from the Affiliated Hospital of Xuzhou Medical University ICU. The flow of patient inclusion and exclusion is depicted in **Figure 1**. From the MIMIC-IV database, 11,781 adult patients admitted to the ICU for the first time with a stay of ≥ 72 hours and a diagnosis of sepsis were screened. Patients were excluded due to malignancy (n=1,587), metastatic tumors (n=191), AIDS (n=78), missing data (n=6,006), non-fulfillment of ARDS criteria (n=995), steroid use within 6 hours of admission (n=143), or outlier data (n=296). Ultimately, 2,485 critically ill patients were included in the internal analysis. Similarly, 346 eligible patients were screened from the external center, with 298 included in the validation cohort after exclusions. The external validation cohort exhibited higher illness severity scores and a greater prevalence of organ dysfunction compared to the derivation cohort. The distinct distribution of GLR between the two cohorts is illustrated in **Supplementary Figure 6**.

**Table 1** compares the demographic and clinical characteristics of 28-day survivors and non-survivors in the MIMIC-IV cohort. Of the 2,485 participants, 58.95% were male, and the average age was 60.66 years. Non-survivors exhibited significantly higher heart rate, respiratory rate, WBC, RBC, platelets, bilirubin, PTT, glucose, BUN, creatinine, FiO_2_, lactate, PEEP, and severity scores (SOFA, SAPS II, APS III), as well as a higher prevalence of severe liver disease and vasoactive drug use (P < 0.05). Conversely, non-survivors had lower temperature, SpO_2_, lymphocyte counts, pH, PaO_2_, and BE, alongside higher rates of heart failure, cerebrovascular disease, renal disease, and AKI (P < 0.05). **Table 2** demonstrates that as GLR levels increased, patients were older, and gradients of metabolic-inflammatory stress and organ damage progressively worsened. Variables including heart rate, respiratory rate, FiO_2_, glucose, Cr, BUN, liver enzymes, bilirubin, lactate, severity scores, and the proportion of severe ARDS all showed a stepwise increase (P < 0.01), while SpO_2_, PaO_2_, pH, BE, lymphocytes, Hb, and HCT decreased (P < 0.001). Q4 patients demonstrated a 28-day mortality of 30.1%, significantly above the 14.2% seen in Q1 (P < 0.001). **Supplementary Tables 1**, **2** details the missing data for both included and excluded variables.

**Table 1:** Baseline characteristics of the study participants.

Variable names	Total (n=2,485)	Survival group (n=1,936)	Non-survival group (n=549)	*P* value
Demographics				
Age(years)	60(48-69)	59(47-69)	62 (52-70)	<0.001
Gender, n(%)				
Male	1465 (58.95)	1145 (59.14)	320 (58.29)	0.756
Female	1020 (41.05)	791 (40.86)	229 (41.71)	
Height(cm)	170 (163-178)	170 (163-178)	170 (163-178)	0.622
Weight(Kg)	92.6 (77-110)	92.9 (77-109.32)	91.4 (77-111.6)	0.105
BMI(Kg/m^2^)	31.76(27.04-37.31)	31.65(27.13-37.1)	32.14(26.68-37.89)	0.089
Comorbidities, n (%)				
Congestive heart failure	402 (16.18)	297 (15.34)	105 (19.13)	0.039
Diabetes	675 (27.16)	520 (26.86)	155 (28.23)	0.559
Chronic pulmonary disease	645 (25.96)	502 (25.93)	143 (26.05)	1.000
Cerebrovascular disease	388 (15.61)	274 (14.15)	114 (20.77)	<0.001
Myocardial infarct	377 (15.17)	281 (14.51)	96 (17.49)	0.100
Renal disease	393 (15.81)	282 (14.57)	111 (20.22)	0.002
Severe liver disease	2179 (87.69)	1771 (91.48)	408 (74.32)	<0.001
AKI	2346 (94.41)	1806 (93.29)	540 (98.36)	<0.001
Vital signs				
Temperature (°C)	36.89 (36.44-37.33)	36.89 (36.44-37.39)	36.78 (36.39-37.17)	<0.001
Heart rate (beats/min)	90 (79-107)	90 (79-106)	94 (81-109)	0.028
Resp rate (beats/min)	20 (16-24)	19.25 (16-24)	21 (18-26)	<0.001
SBP (mmHg)	119 (104-136)	119 (104-136)	118 (102-136)	0.143
DBP (mmHg)	67 (56-78)	67 (56-78)	67 (55-81)	0.676
MBP (mmHg)	82 (70-94)	82 (70-94)	82 (70-94)	0.309
SpO_2_ (%)	98 (95-100)	98 (95-100)	97 (94-100)	<0.001
Laboratory tests				
WBC (K/uL)	12.6 (8.6-17.7)	12.4 (8.6-17.3)	13.3 (8.6-18.7)	0.005
Hemoglobin (g/L)	10.7 (8.9-12.5)	10.7 (9-12.5)	10.5 (8.7-12.6)	0.435
Hematocrit(L/L)	32.4 (27.3-37.9)	32.45 (27.6-37.8)	32.2 (26.6-38.7)	0.933
RDW (%)	14.5 (13.4-16)	14.3 (13.4-15.7)	15.2 (14-17.6)	<0.001
Lymphocytes(10^9^/L)	0.95 (0.59-1.48)	1.02 (0.62-1.55)	0.83 (0.5-1.29)	<0.001
GLR	3.19 (2.42-4)	3.09 (2.34-3.9)	3.61 (2.81-4.35)	<0.001
PLT (K/uL)	180 (118-251)	184 (124.75-251)	162 (92-247)	<0.001
Creatinine(mg/dl)	1 (0.7-1.7)	1 (0.7-1.5)	1.4 (0.9-2.4)	<0.001
BUN(mg/dl)	20 (13-34)	19 (13-30)	29 (17-45)	<0.001
ALT(U/L)	33 (19-71)	31 (19-66)	39 (23-97)	0.702
AST(U/L)	54 (30-118)	51 (29-105)	73 (39-181)	0.309
Bilirubin(μmol/L)	0.7 (0.4-1.5)	0.6 (0.4-1.2)	0.9 (0.4-3.7)	<0.001
PTT(s)	31.3 (27.4-39)	30.7 (27-37.1)	35 (29.1-47.4)	<0.001
Glucose(mg/dl)	151 (123-196)	147.5 (121-188)	166 (132-218)	<0.001
PH	7.35 (7.27-7.41)	7.35 (7.28-7.41)	7.33 (7.25-7.39)	<0.001
FiO_2_(%)	50 (40-70)	50 (40-60)	50 (40-80)	<0.001
PaO_2_(mmHg)	98 (62-194)	105 (66-215.25)	80 (50-134)	<0.001
PaCO_2_(mmHg)	42 (36-49)	42 (36-49)	42 (35-49)	0.736
SpO_2_(%)	98 (95-100)	98 (95-100)	97 (94-100)	<0.001
Lactate(mmol/L)	1.9 (1.2-3)	1.7 (1.2-2.8)	2.3 (1.5-3.8)	<0.001
Calcium(mg/dl)	1.11 (1.04-1.17)	1.11 (1.04-1.17)	1.1 (1.02-1.17)	0.164
BE(mmol/L)	-1 (-6-1)	-1 (-5-1)	-3 (-7-0)	<0.001
Ventilation settings				
Peep(cmH_2_O)	5 (5-10)	5 (5-8)	5 (5-10)	0.001
VT(ml/kg)	458.38 (397.92-525)	460.4 (399.98-525.99)	450.15 (386.29-520.22)	0.240
Score				
APSIII	53 (38-73)	50 (37-67)	69 (51-90)	<0.001
SAPSII	53 (38-73)	50 (37-67)	69 (51-90)	<0.001
SOFA score	40 (32-50)	38 (31-48)	48 (39-59)	<0.001
Sirs				
0	10 (0.40)	8 (0.41)	2 (0.36)	0.040
1	122 (4.91)	101 (5.22)	21 (3.83)	
2	515 (20.72)	420 (21.69)	95 (17.30)	
3	1051 (42.29)	791 (40.86)	260 (47.36)	
4	787 (31.67)	616 (31.82)	171 (31.15)	
ARDS classification				
Mild	630 (25.35)	510 (26.34)	120 (21.86)	<0.001
Moderate	1063 (42.78)	846 (43.70)	217 (39.53)	
Severe	792 (31.87)	580 (29.96)	212 (38.62)	
Treatments, n(%)				
Vasoactive	1667 (67.08)	1254 (64.77)	413 (75.23)	<0.001

BMI, Weight (kg) ÷ height (m)²; SBP, Systolic blood pressure; DBP, Diastolic blood pressure; MBP, Mean blood pressure; SpO2, Blood oxygen saturation; WBC, White blood cells; RDW, Red blood cell distribution width; PLT, Platelet; BUN, Blood urea nitrogen; GLR, log_2_[admission ICU blood glucose(mg/dl)/(18*lymphocytes(mmol/L)]; ALT, Alanine aminotransferase; AST, Aspartate aminotransferase; PTT, Partial thromboplastin time; PH, Potential of hydrogen; FiO2, Fraction of inspiration O2; PaO2, Partial pressure of oxygen in arterial blood; PaCO2, Partial pressure of carbon dioxide; SPO2, Saturation of peripheral oxygen; BE, Base excess; PPEP, Positive end-expiratory pressure; VT, Ventricular tachycardia; SOFA, Sepsis-related organ failure assessment score; SAPSII, Simplified acute physiology score II; APSIII, Acute physiology score III; Sirs, Systemic inflammatory response syndrome; AKI, Acute kidney injury; ARDS, Acute respiratory distress syndrome, mild:PaO2/FiO2<100mmHg;moderate:100mmHg<PaO2/FiO2<200mmHg;severe:200mmHg<PaO2/FiO2<300mmHg.

**Table 2:** Baseline characteristics according to GLR quartiles.

Variable names	Total (n=2,485)	Q1 (n=622)	Q2 (n=621)	Q3 (n=621)	Q4 (n=621)	*P*
Demographics						
Age(years)	60 (48-69)	59 (46-70)	58 (46-68)	60 (50-68)	63 (52-71)	<0.001
Gender, n(%)						
Male	1465 (58.95)	385 (61.90)	360 (57.97)	363 (58.45)	357 (57.49)	0.378
Female	1020 (41.05)	237 (38.10)	261 (42.03)	258 (41.55)	264 (42.51)	
Height(cm)	170 (163-178)	170 (163-178)	170 (163-178)	170 (163-178)	170 (163-178)	0.142
Weight(kg)	92.6 (77-110)	92.75 (79.43-108.88)	93.3 (76.8-111.2)	93.3 (76-110)	90.8 (77.1-109.2)	0.513
BMI(Kg/m^2^)	31.77(27.04-37.31)	31.79 (27.4-36.71)	32.13 (27.09-37.89)	31.54 (26.76-37.48)	31.78 (27.05-36.93)	0.734
Comorbiditiesn, n(%)						
Congestive heart failure	402 (16.18)	93 (14.95)	94 (15.14)	110 (17.71)	105 (16.91)	0.474
Diabetes	675 (27.16)	144 (23.15)	161 (25.93)	173 (27.86)	197 (31.72)	0.007
Chronic pulmonary disease	645 (25.96)	162 (26.05)	153 (24.64)	154 (24.80)	176 (28.34)	0.418
Cerebrovascular disease	388 (15.61)	105 (16.88)	108 (17.39)	90 (14.49)	85 (13.69)	0.205
Myocardial infarct	377 (15.17)	99 (15.92)	93 (14.98)	91 (14.65)	94 (15.14)	0.937
Renal disease	393 (15.81)	91 (14.63)	85 (13.69)	100 (16.10)	117 (18.84)	0.07
Severe liver disease	306 (12.31)	61 (9.81)	84 (13.53)	79 (12.72)	82 (13.20)	0.171
AKI	2346 (94.41)	574 (92.28)	578 (93.08)	593 (95.49)	601 (96.78)	0.002
Vital signs						
Temperature (°C)	36.9 (36.4-37.3)	36.8 (36.4-37.3)	36.8 (36.4-37.3)	36.9 (36.4-37.3)	36.9 (36.4-37.3)	0.862
Heart rate (beats/min)	90 (79-107)	86 (78-101)	90 (80-107)	93 (80-110)	94 (79-110)	<0.001
Resp rate (beats/min)	20 (16-24)	18 (15-22)	20 (16-24)	20 (16-25)	21 (17-26)	<0.001
SBP (mmHg)	119 (104-136)	119 (104-134.75)	118 (104-135)	121 (105-138)	117 (101-136)	0.11
DBP (mmHg)	67 (56-78)	66 (56-77)	67 (56-79)	68 (57-82)	66 (56-77)	0.04
MAP (mmHg)	82 (70-94)	82 (71-93)	82 (70-94)	83 (71-97)	80 (69-92)	0.077
SpO_2_ (%)	98 (95-100)	99 (96-100)	98 (95-100)	98 (94-100)	97 (94-100)	<0.001
Laboratory tests						
WBC (K/uL)	12.6 (8.6-17.7)	13.5 (10.1-17.5)	13.5 (9.6-18.3)	12.6 (8.7-18.5)	10.6 (6.3-15.7)	<0.001
Hemoglobin (g/L)	10.7 (8.9-12.5)	10.1 (8.6-11.9)	10.7 (8.9-12.7)	10.9 (9.1-12.8)	10.8 (9.1-12.6)	<0.001
Hematocrit(L/L)	32.4 (27.3-37.9)	30.7 (26.35-36.2)	32.4 (27-38.2)	33.1 (27.8-38.5)	33.3 (28.6-38.4)	<0.001
RDW (%)	14.5 (13.4-16)	14.2 (13.3-15.8)	14.5 (13.4-16.2)	14.6 (13.6-15.9)	14.6 (13.6-16.2)	0.086
Lymphocytes(109/L)	0.91 (0.59-1.48)	1.85 (1.49-2.29)	1.16 (0.93-1.43)	0.77 (0.62-0.97)	0.4 (0.27-0.55)	<0.001
PLT (K/uL)	180 (118-251)	173.5 (125-243)	195 (125-259)	188 (116-262)	166 (106-237)	0.003
Creatinine(mg/dl)	1 (0.7-1.7)	0.9 (0.7-1.3)	1 (0.7-1.6)	1.1 (0.8-1.8)	1.2 (0.8-2.2)	<0.001
BUN(mg/dl)	20 (13-34)	17 (12-25)	19 (13-31)	20 (14-35)	26 (16-46)	<0.001
ALT(U/L)	33 (19-71)	29 (17-54.75)	32 (19-67)	36 (20-75)	38 (21-90)	0.011
AST(U/L)	54 (30-118)	48 (28-90)	52 (30-127)	57 (33-134)	55 (31-124)	0.001
Bilirubin total(μmol/L)	0.7 (0.4-1.5)	0.6 (0.4-1.3)	0.7 (0.4-1.4)	0.6 (0.4-1.4)	0.7 (0.4-1.8)	0.017
PTT(s)	31.3 (27.4-39)	31 (27.5-37.6)	30.9 (27-38.5)	31.5 (27.5-39.9)	31.8 (27.5-39.8)	0.44
Glucose(mg/dl)	151 (123-196)	123 (105-141.75)	144 (122-176)	166 (136-210)	196 (152-241)	<0.001
PH	7.35 (7.27-7.41)	7.38 (7.32-7.42)	7.36 (7.28-7.41)	7.33 (7.25-7.4)	7.32 (7.24-7.39)	<0.001
FiO_2_(%)	50 (40-70)	50 (30-60)	50 (40-70)	50 (40-70)	50 (40-80)	<0.001
PaO_2_(mmHg)	98 (62-194)	135.5 (73-292.75)	95 (62-185)	94 (61-167)	86 (56-149)	<0.001
PaCO_2_(mmHg)	42 (36-49)	42 (37-47)	42 (36-48)	42 (36-50)	43 (35-52)	0.001
Lactate(mmol/L)	1.9 (1.2-3)	1.6 (1.1-2.6)	1.8 (1.2-2.9)	1.9 (1.3-3.3)	2.1 (1.3-3.4)	<0.001
Calcium(mg/dl)	1.11 (1.04-1.17)	1.12 (1.06-1.18)	1.11 (1.04-1.17)	1.1 (1.03-1.16)	1.09 (1.02-1.16)	0.006
BE(mmol/L)	-1 (-6-1)	0 (-3-2)	-1 (-5-0)	-2 (-7-1)	-3 (-7-0)	<0.001
Ventilation settings						
Peep(cmH_2_O)	5 (5-10)	5 (5-8)	5 (5-10)	5 (5-10)	5 (5-10)	<0.001
VT(ml)	458.38 (397.92-525)	463.99 (401.76-528.76)	459.06 (400.54-530)	455.84 (393.64-525.83)	453.5 (397.41-512.5)	0.545
Disease severity score						
SAPSII score	40 (32-50)	37 (30-46)	39 (31-49)	40 (33-50)	44 (35-56)	<0.001
APSIII score	53 (38-73)	44 (32-64)	52 (38-72)	56 (41-74)	60 (47-82)	<0.001
SOFA score	2 (0-4)	2 (0-4)	1 (0-4)	2 (0-5)	2 (0-4)	0.264
Sirs						
0	10 (0.40)	1 (0.16)	4 (0.64)	3 (0.48)	2 (0.32)	0.008
1	122 (4.91)	38 (6.11)	23 (3.70)	35 (5.64)	26 (4.19)	
2	515 (20.72)	156 (25.08)	130 (20.93)	120 (19.32)	109 (17.55)	
3	1051 (42.29)	240 (38.59)	286 (46.05)	266 (42.83)	259 (41.71)	
4	787 (31.67)	187 (30.06)	178 (28.66)	197 (31.72)	225 (36.23)	
ARDS classification						
Mild	630 (25.35)	187 (30.06)	160 (25.76)	144 (23.19)	139 (22.38)	<0.001
Moderate	1063 (42.78)	288 (46.30)	266 (42.83)	264 (42.51)	245 (39.45)	
Severe	792 (31.87)	147 (23.63)	195 (31.40)	213 (34.30)	237 (38.16)	
Treatments, n(%)						
Vasoactive	1667 (67.08)	408 (65.59)	407 (65.54)	412 (66.34)	440 (70.85)	0.142
Outcomes						
28-Day death	549 (22.09)	88 (14.15)	118 (19.00)	156 (25.12)	187 (30.11)	<0.001

BMI, Weight (kg) ÷ height (m)²; SBP, Systolic blood pressure; DBP, Diastolic blood pressure; MBP, Mean blood pressure; SpO2, Blood oxygen saturation; WBC, White blood cells; RDW, Red blood cell distribution width; PLT, Platelet; BUN, Blood urea nitrogen; GLR, log_2_[admission ICU blood glucose(mg/dl)/(18*lymphocytes(mmol/L)]; ALT, Alanine aminotransferase; AST, Aspartate aminotransferase; PTT, Partial thromboplastin time; PH, Potential of hydrogen; FiO2, Fraction of inspiration O2; PaO2, Partial pressure of oxygen in arterial blood; PaCO2, Partial pressure of carbon dioxide; SPO2, Saturation of peripheral oxygen; BE, Base excess; PPEP, Positive end-expiratory pressure; VT, Ventricular tachycardia; SOFA, Sepsis-related organ failure assessment score; SAPSII, Simplified acute physiology score II; APSIII, Acute physiology score III; Sirs: Systemic inflammatory response syndrome; AKI, Acute kidney injury; ARDS, Acute respiratory distress syndrome.

### 3.2 Association between GLR and clinical outcomes

**Figure 2:**
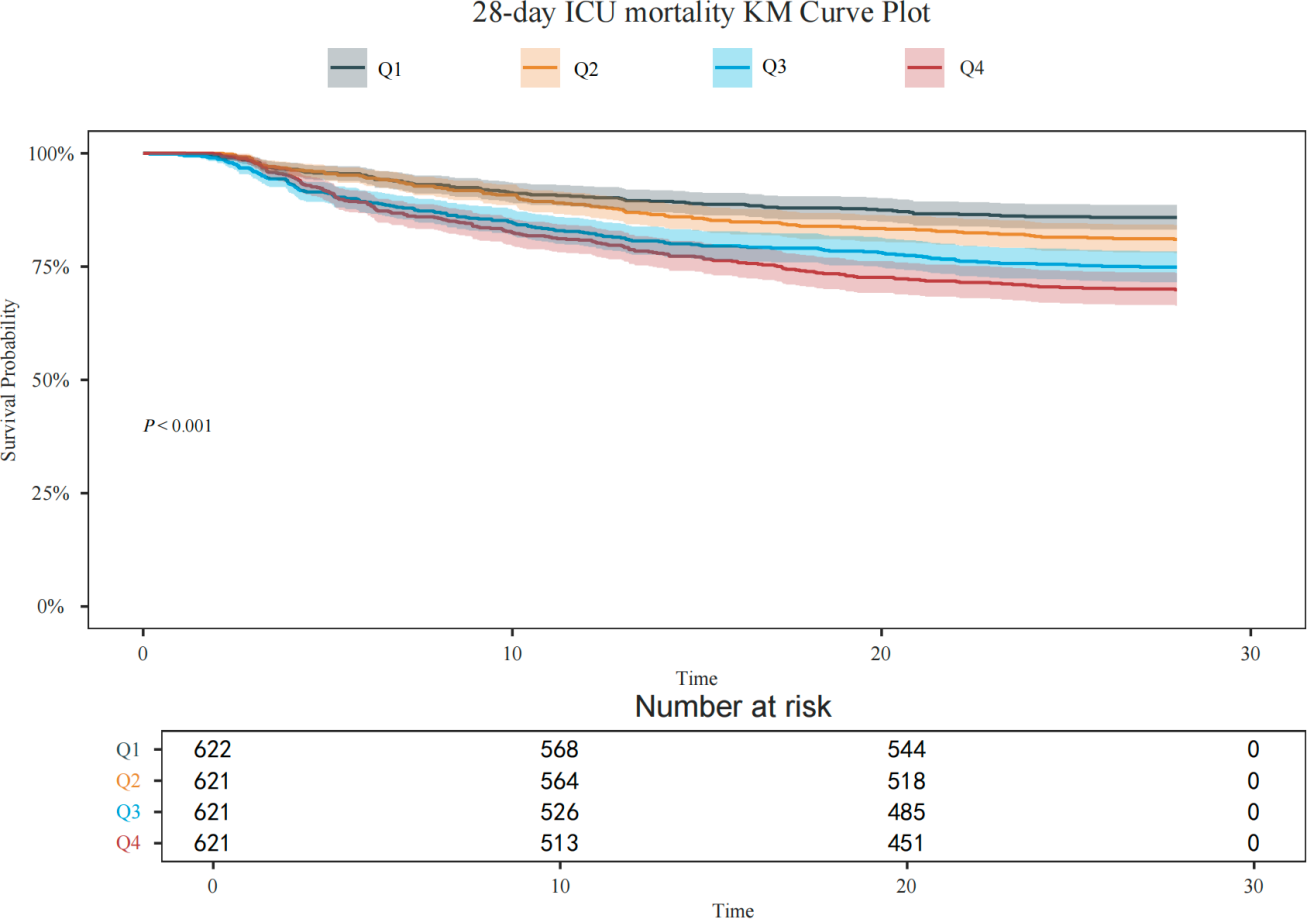
Kaplan-Meier survival curves for 28-day ICU mortality stratified by GLR quartiles. The shaded areas represent 95% confidence intervals. The difference in survival rates among groups was statistically significant (Log-rank test *P* < 0.001).

Kaplan–Meier survival curves revealed significant differences in prognosis among the four groups, with the log-rank test confirming statistical significance (P < 0.001). The 28-day ICU survival probability declined significantly in a stepwise manner with increasing GLR levels. Individuals in the highest GLR quartile (Q4) exhibited the lowest cumulative survival, whereas those in the lowest quartile (Q1) showed the most favorable outcomes. These results suggest a strong link between elevated GLR levels and adverse clinical outcomes (**Figure 2**).

**Figure 3:**
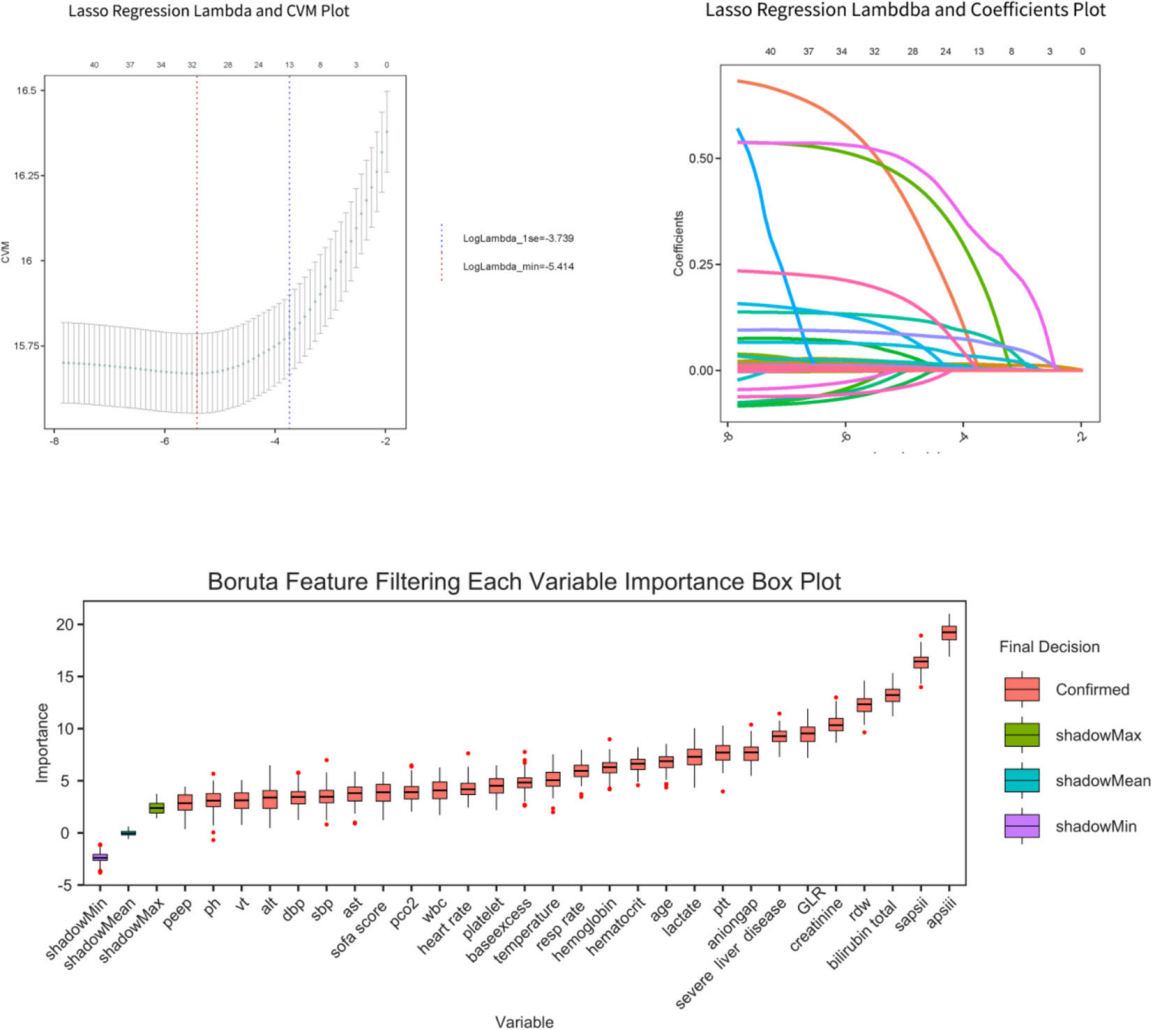
Lasso regression and Boruta algorithm identified predictive variables associated with 28-day mortality following ICU admission.

**Figure 4:**
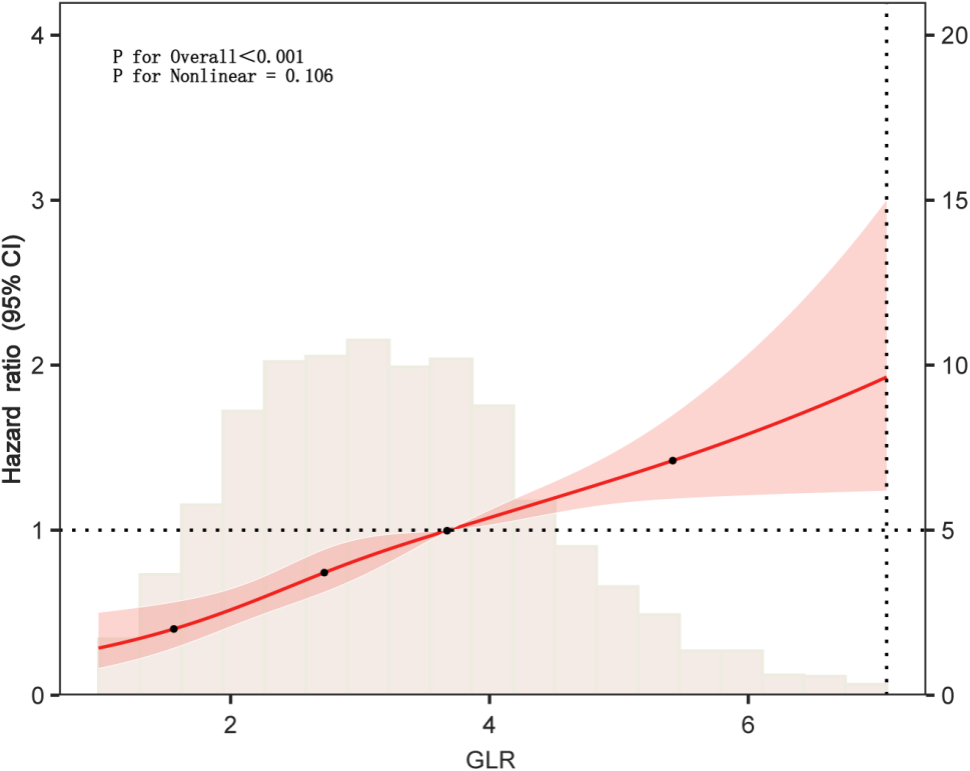
Restricted cubic spline analysis of the association between GLR and the risk of 28-day ICU death in Patients with ARDS Complicating the Acute Phase of Sepsis.

GLR showed moderate-to-strong positive correlations with lactate, SOFA, SAPS II, APS III, total bilirubin, creatinine, BUN, and FiO_2_ (|r| = 0.40~0.70, P < 0.001), and moderate-to-strong negative correlations with lymphocytes, PaO_2_, pH, and BE (|r| = –0.30~–0.55, P < 0.001). This suggests GLR serves as a composite marker reflecting inflammatory-metabolic stress and multi-organ dysfunction. Weak correlations (|r|<0.3) with ALT, AST, age, heart rate, and respiratory rate indicated manageable collinearity risk and independent informational value (**Supplementary Figure 1**). To balance model robustness and clinical interpretability, LASSO regression combined with the Boruta algorithm identified ten key variables as predictors of 28-day mortality: age, anion gap, APS III, hematocrit, lactate, PTT, RDW, SAPS II, GLR, and severe liver disease (**Figure 3**). VIF screening revealed moderate collinearity between SAPS II and APS III (**Supplementary Table 3**). However, based on clinical relevance—representing overall physiological reserve versus acute organ dysfunction, respectively—both were retained. Multivariate Cox regression analyses consistently showed that higher GLR levels were linked to increased 28-day ICU mortality in both the internal and external cohorts (**Table 3**). When treated as a continuous variable, each one-unit increase in GLR corresponded to a 17% higher risk of mortality in the unadjusted model (HR = 1.17, 95% CI: 1.120–1.230; P < 0.001). This relationship remained statistically significant after full covariate adjustment (HR = 1.13, 95% CI: 1.053–1.211; P < 0.001). This predictive value was even more pronounced in the external cohort (Adjusted HR = 1.845, 95% CI: 1.452–2.345, P < 0.001). When analyzed as a categorical variable, Q4 showed the highest risk in the unadjusted model (HR = 2.326, 95% CI: 1.806–2.997) and remained the highest risk group after full adjustment (Model 3: HR = 1.416, 95% CI: 1.088–1.842, P = 0.013). Q3 also demonstrated a significant independent risk effect (HR = 1.399, P = 0.013) (**Table 3**, **Supplementary Table 4**). A significant linear trend of increasing mortality risk across GLR quartiles was observed (P for trend < 0.01). RCS analysis (**Figure 4**) indicated no statistically significant non-linear relationship between GLR and 28-day mortality (P for non-linearity > 0.05).

**Table 4:** Prognostic accuracy of the SAPS II, APS III, and GLR.

	AUC (95%CI)	Sensitivity (95%CI)	Specificity (95%CI)	PPV (95%CI)	NPV (95%CI)
28-day ICU mortality					
SAPSII	0.678(0.640-0.700)	0.730(0.480-0.800)	0.530(0.470-0.770)	0.230(0.220-0.2900)	0.910(0.880-0.920)
SAPSII+GLR	0.696(0.660-0.710)	0.610(0.45-0.730)	0.610(0.450-0.730)	0.250(0.220-0.30)	0.910(0.890-0.930)
APSIII	0.694(0.670-0.720)	0.610(0.480-0.750)	0.610(0.480-0.750)	0.350(0.310-0.420)	0.86(0.840-0.890)
APSIII + GLR	0.708(0.680-0.730)	0.690(0.620-0.780)	0.630(0.540-0.690)	0.340(0.3-0.370)	0.88(0.860-0.900)

APSIII, Acute physiology score III; GLR, Glucose to lymphocyte ratio; SAPSII, Simplified acute physiology score II.

### 3.3 Subgroup analysis

**Figure 5:**
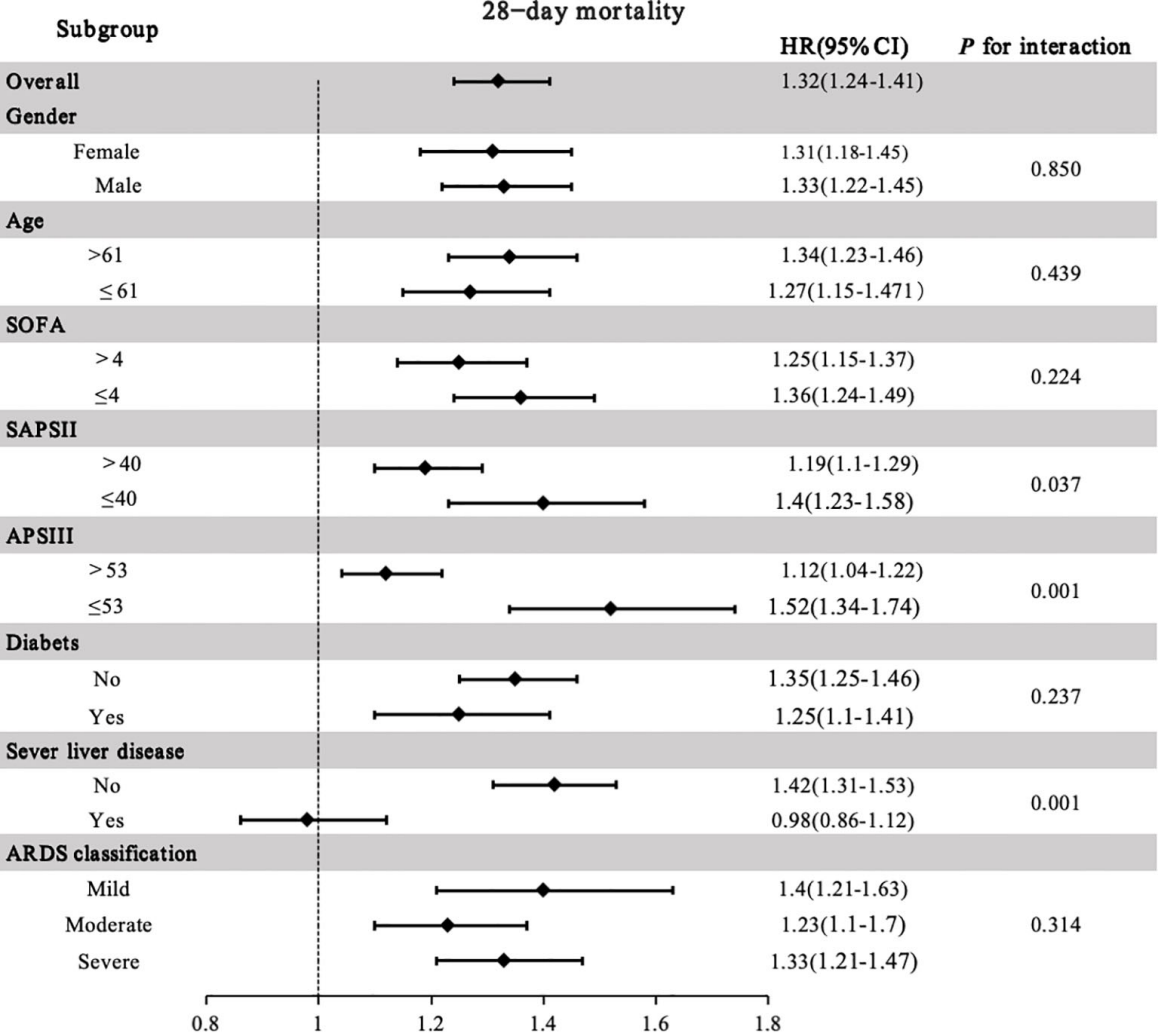
Forest plot of subgroup analyses for the association between GLR and 28-day ICU mortality.

The association between GLR and 28-day ICU mortality was further assessed through subgroup analyses to confirm its robustness (**Figure 5**). The deleterious effect of elevated GLR on survival remained consistent across most strata, including age, gender, SOFA score, diabetes, and ARDS severity, with no significant interactions observed (all P for interaction > 0.05). However, significant interactions were identified for disease severity scores (SAPS II, APS III) and severe liver disease. The prognostic strength of GLR was significantly attenuated in patients with greater illness severity. While GLR was a strong predictor in patients with SAPS II ≤ 40 (HR 1.40, 95% CI: 1.23–1.58), its value decreased in those with SAPS II > 40 (HR 1.19, 95% CI: 1.10–1.29; P for interaction = 0.037). A similar pattern was observed for APS III (P for interaction = 0.001). In patients with severe liver disease, the association between GLR and mortality was no longer statistically significant (HR 0.98, 95% CI: 0.86–1.12), whereas it remained strong in those without severe liver disease (HR 1.42, 95% CI: 1.31–1.53; P for interaction = 0.001), indicating heterogeneity between the subgroups.

### 3.4 Predictive performance and additional prognostic effect of GLR

**Figure 6:**
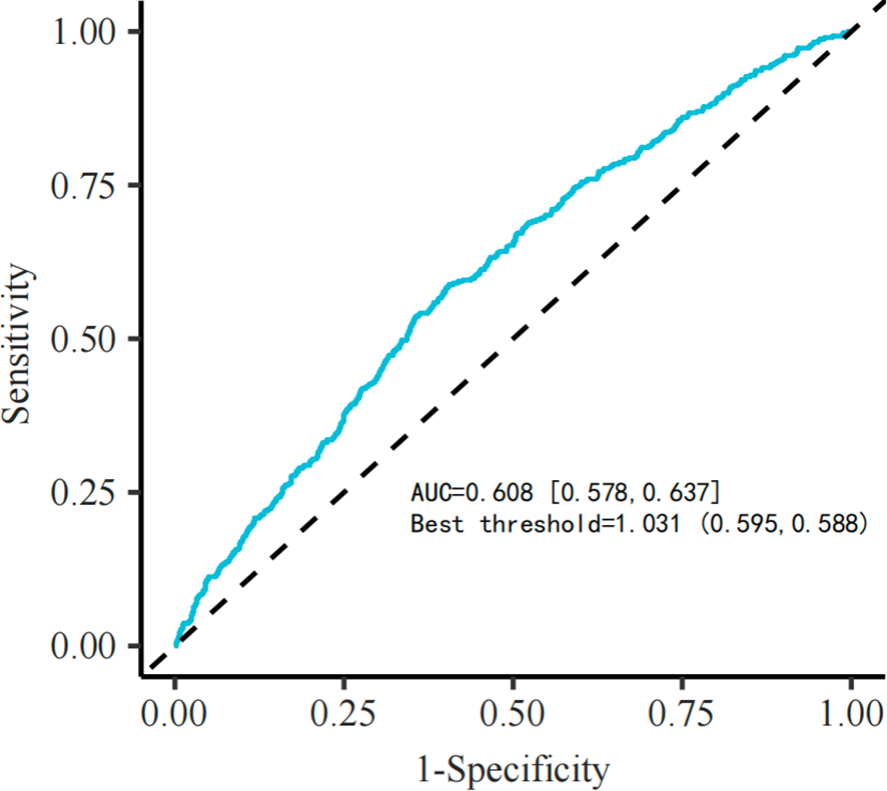
Internal data GLR ROC curve.

**Figure 7:**
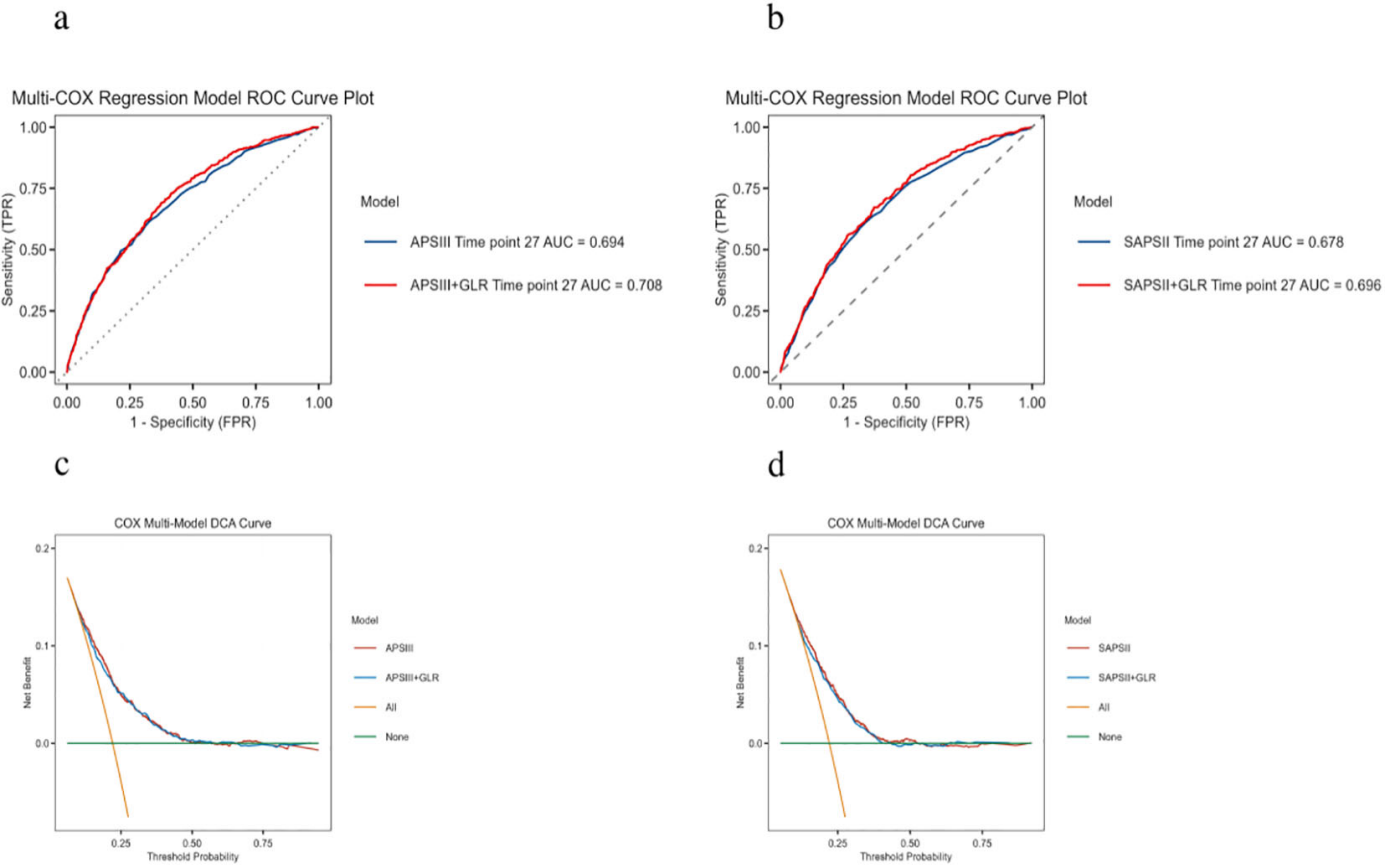
ROC and DCA curve analysis of the incremental effect of GLR on 28-day- all-cause mortality. **(a)** APSIII score + GLR ROC curve. **(b)** SAPSII score + GLR ROC curve **(c)** APSIII score + GLR DCA curve. **(d)** SAPSII score + GLR DCA curve.

In the internal dataset, GLR as a standalone factor (optimal cutoff = 1.031) demonstrated moderate predictive value for 28-day outcomes (AUC = 0.608), with similar performance in the external dataset (AUC = 0.695, optimal cutoff = 1.584) (**Figure 6**, **Supplementary Figure 2**). Due to suboptimal standalone accuracy, GLR was integrated with existing prognostic models. Results showed that GLR enhanced the predictive capability of both scoring systems (**Table 4**; **Figure 7**): the AUC for APS III increased from 0.694 (0.670–0.720) to 0.708 (0.680–0.730), and for SAPS II from 0.678 (0.640–0.700) to 0.696 (0.660–0.710). This incremental value was maintained in the external dataset (**Supplementary Figure 3**). DCA curves demonstrated that prognostic scores combined with GLR yielded superior clinical net benefit compared to the scoring models alone.

**Table 3:** Unadjusted and multivariate Cox regression analyses were employed to assess 28-day ICU mortality.

Characteristic	Model 1		Model 2		Model 3	
	HR (95% CI)	*P*-value	HR (95% CI)	*P*-value	HR (95% CI)	*P*-value
28-day mortality rate						
GLR (for each additional unit)	1.17 (1.12 ~ 1.23)	<0.001	1.233 (1.152~ 1.321)	<0.001	1.130 (1.053 ~ 1.211)	0.001
GLR (quartiles)						
Q1	1.00 (Reference)		1.00 (Reference)		1.00 (Reference)	
Q2	1.365 (1.036 ~ 1.799)	0.027	1.211 (0.917 ~ 1.598)	0.177	1.096 (0.829 ~ 1.448)	0.520
Q3	1.907 (1.469 ~ 2.477)	<0.001	1.599(1.228 ~ 2.083)	<0.001	1.399 (1.072 ~ 1.826)	0.013
Q4	2.326 (1.806 ~ 2.997)	<0.001	1.829 (1.414 ~ 2.366)	<0.001	1.416 (1.088 ~ 1.842)	0.013
P for trend		<0.001		<0.001		0.001

Model 1: no other covariates were adjusted.

Model 2: Adjust for age, aniongap, bilirubin, hematocrit, lactate, PTT, RDW.

Model 3: Adjust for age, aniongap, bilirubin, hematocrit, lactate, PTT, RDW, APSIII, SAPSII, severe liver disease.

HR, Hazard ratio; CI, Confidence interval; PTT, Partial thromboplastin time; RDW, Red blood cell distribution width; APSIII, Acute physiology score III; SAPSII, Simplified acute physiology score II.

## 4 Discussion

To our knowledge, this is the first study to investigate the relationship between the GLR assessed at early ICU admission and short-term mortality in patients with sepsis-associated ARDS. The prognostic value of GLR in this high-risk population was further confirmed using external validation data. The key findings indicate that GLR is an important independent predictor of 28-day mortality in these critically ill patients; after adjusting for potential confounders, elevated GLR remained significantly associated with increased mortality risk, with each unit increase corresponding to a 13% higher risk of death (HR: 1.13, 95% CI: 1.053–1.211). RCS analysis showed a linear “dose–response” relationship between GLR and 28-day ICU mortality (P for non-linearity = 0.106). Furthermore, incorporating GLR into existing risk scoring models (such as APS III and SAPS II) significantly enhanced their predictive performance for clinical outcomes. It is worth noting that a stronger association between GLR and 28-day ICU mortality was observed in the external validation cohort compared with the MIMIC-IV derivation cohort. This discrepancy may be attributed to several factors: (i) Different patient characteristics, as the external cohort had higher overall severity scores and a higher prevalence of organ dysfunction; (ii) The smaller sample size of the external cohort (n=298), which can lead to wider confidence intervals and potentially inflated effect estimates; and (iii) Potential regional or treatment differences, given that the external cohort was from a single center in China, while the MIMIC-IV cohort is U.S.-based. This observation highlights the importance of external validation and suggests that the predictive strength of GLR may vary across populations with differing baseline risks and clinical management practices.

In oncology, a meta-analysis encompassing 22 studies with a total of 9,472 patients indicated that higher pre-treatment GLR levels were independently linked to worse overall survival (OS) across multiple solid tumor types, including hepatocellular carcinoma, breast, pancreatic, and colorectal cancers (HR = 1.48, 95% CI: 1.34–1.63). These associations remained consistent in subgroup analyses, supporting the robustness of the findings (Liu et al., 2024). In cardiovascular diseases, Christos Kofos et al. reported that PLR and GLR have prognostic significance in patients with acute coronary syndrome (ACS), finding that high GLR is associated with heightened inflammation and adverse cardiovascular events, increasing mortality risk (OR: 1.006, 95% CI: 1.003–1.008), and its predictive performance is comparable to the platelet-to-lymphocyte ratio (PLR); the study also highlighted that incorporating GLR into traditional risk scoring systems such as GRACE significantly improves predictive accuracy (Kofos et al., 2025). A recent large prospective cohort study indicated that GLR can be applied for survival prediction in healthy populations; in middle-aged and elderly individuals without acute illness, elevated baseline GLR levels were independently associated with long-term mortality risk, especially from cardiovascular and cerebrovascular diseases, reflecting a chronic, low-grade “metabolic-inflammation” (metaflammation) imbalance that may contribute to atherosclerosis, insulin resistance, and neurodegeneration (Lyu et al., 2025). Additionally, studies in patients with atherosclerotic cardiovascular disease (ASCVD) demonstrated that higher GLR is independently associated with increased long-term mortality, establishing GLR as a key indicator of adverse prognosis in ASCVD patients (Lei et al., 2025). Another study showed that in patients undergoing percutaneous coronary intervention (PCI) for coronary artery disease, GLR, regardless of the timing of glucose measurement, robustly predicted long-term adverse outcomes, and in STEMI patients undergoing emergent PCI, elevated admission GLR strongly predicted all-cause and cardiovascular mortality (Liu et al., 2024). Gang Wu et al. reported a study based on the MIMIC-IV database showing that high GLR was significantly associated with both short- and long-term mortality in critically ill heart failure patients (HR 1.57, 95% CI 1.45–1.70; HR 1.48, 95% CI 1.40–1.56), improving prognostic accuracy (Wu et al., 2025). Cai S et al. analyzed data from 10,118 sepsis patients in the MIMIC-IV database and reported that elevated GLR was significantly associated with increased in-hospital mortality (adjusted HR = 1.02, 95% CI: 1.01–1.03). Notably, restricted cubic spline (RCS) analysis indicated that the relationship between GLR and in-hospital mortality was nonlinear (Cai et al., 2022). Another study indicated that admission GLR was an independent predictor of 28-day mortality in critically ill ARDS patients, and after adjusting for key confounders (PaO_2_/FiO_2_, etiology, and SOFA score), the association between GLR and mortality remained significant (OR = 1.67, 95% CI: 1.26–2.22) (Zhang and Zhang, 2022). These earlier investigations support the observations made in our high-risk cohort. To our knowledge, this is the first study exploring the prognostic value of GLR for short-term mortality in sepsis-induced ARDS, emphasizing the pivotal contribution of metabolic–immune dysregulation to ARDS pathophysiology.

In subgroup analyses, we observed that GLR was more prominent in patients with lower disease severity (low SAPS II and APS III scores) and in those without severe liver disease, with a more pronounced effect on increased mortality risk, whereas in patients with severe liver disease, GLR was not associated with mortality risk (HR = 0.98, 95% CI: 0.86–1.12), indicating significant heterogeneity in the predictive effect of GLR on 28-day mortality across these subgroups. This effect may be explained by the observation that, in patients with very severe disease (SAPS II > 40 or APS III > 53), prognosis is likely determined primarily by the severity of underlying disease, the extent of multi-organ failure, and other irreversible pathophysiological processes, such that minor variations in GLR cannot significantly alter outcomes in these high-risk patients. Conversely, in patients with relatively lower disease severity, GLR may serve as a more sensitive early warning indicator, as these patients have not yet progressed to end-stage multi-organ failure, and elevated GLR may more promptly reflect internal immunometabolic imbalance, indicating a higher risk of progression to critical illness or death (Zhou et al., 2025). Liu and Hu (2023)) found that in patients with acute myocardial infarction (AMI), elevated GLR was independently associated with mortality, and this association was particularly pronounced in patients with SOFA scores <6, consistent with our findings. Similar studies have shown that combining other inflammatory markers, such as NLR, with APS-III and SOFA can improve predictive accuracy, and in lower score ranges (SOFA <5), NLR has a more significant effect on increased mortality risk (Chen et al., 2025), aligning with the results of our study. Furthermore, in critically ill patients with severe liver disease, there was no statistically significant association between GLR and 28-day mortality, possibly because the liver, as a key organ of the metabolic-immune axis, strongly influences GLR values (Zhang et al., 2024). In cases of severe hepatic decompensation, glucose metabolism is profoundly dysregulated, and immune function, particularly lymphocytes, becomes depleted and dysfunctional (Guo et al., 2025), reducing the specificity of GLR as a biomarker for acute injury in sepsis-induced ARDS and limiting its prognostic utility in this specific patient subgroup.

This study found that GLR is significantly associated with short-term mortality in patients with sepsis-induced ARDS; however, due to the retrospective design, causality cannot be established. The relatively complex pathophysiological mechanism primarily involves a sharp decline in immune cell numbers and functional dysregulation, which is considered a key factor in increased risk of secondary infection and mortality in sepsis patients (Cao et al., 2019). In patients with sepsis and ARDS, stress-induced elevations in blood glucose levels are frequently observed. This response results from sympathetic nervous system activation and elevated secretion of stress-related hormones, including cortisol and catecholamines, which promote insulin resistance, accelerate glycogenolysis and gluconeogenesis, and ultimately lead to elevated blood glucose levels (Liu et al., 2024; Mehdi et al., 2025). Hyperglycemia can significantly suppress immune function, trigger cytokine storms, promote oxidative stress, increase the risk of infection dissemination and organ dysfunction, and amplify pulmonary inflammation and delay epithelial repair via pathways such as TLR4-NF-κB, thereby worsening ARDS and increasing mortality risk in sepsis patients (Fan et al., 2013; Zhang et al., 2023; Yan et al., 2024). Second, during the early stage of sepsis, a large number of lymphocytes, particularly T lymphocytes, undergo apoptosis or migrate to lymphoid tissues and sites of infection, causing a sharp decline in peripheral blood lymphocyte counts; as lymphocytes play a central role in immune surveillance, their reduction weakens host defense against pathogens, accelerating sepsis progression and impairing overall immune function (Liu et al., 2021; Zhang et al., 2024). Lymphocyte depletion leads to immunosuppression, making patients more susceptible to secondary infections, which may progress to persistent inflammation-immunosuppression and catabolism syndrome (PICS), eventually causing multi-organ failure and death (Wang et al., 2024; Gao et al., 2025). In summary, GLR, as a composite indicator, can more comprehensively reflect the severity of immunometabolic imbalance in patients with sepsis-induced ARDS and serves as an effective marker for predicting the complex pathophysiological state in critically ill patients.

The main strength of this study lies in its novel investigation of the association between GLR and short-term mortality in a high-risk subgroup of patients with sepsis-induced ARDS, using a large multicenter database with a sufficient sample size and validated with an external dataset, thereby enhancing the robustness and generalizability of the findings. Second, in terms of clinical application, we confirmed that GLR is an independent prognostic marker. However, its standalone predictive accuracy was modest (AUC ~0.6), indicating that it should not be used in isolation for clinical decision-making. Its primary value lies in its ability to complement existing tools. We demonstrated that integrating GLR into critical illness scoring systems such as APS III and SAPS II can improve their predictive performance for 28-day mortality. While the incremental improvement in the AUC was modest, this finding suggests that GLR captures a distinct aspect of pathophysiological stress—namely, immunometabolic dysfunction—that is not fully accounted for by traditional physiology-based scores. Furthermore, GLR is a low-cost and readily available biomarker, offering practical advantages in resource-limited settings where complex scoring systems may not be immediately calculable. Third, the subgroup analyses revealed heterogeneity across different patient populations and, for the first time, identified SAPSII score, APSIII score, and severe liver disease as modifiers of the predictive effect of GLR, providing guidance for precise prognostic assessment in patients with sepsis-induced ARDS and strengthening the reliability and rigor of the study conclusions. Nevertheless, this study has several limitations. First, it only demonstrates an association between GLR and mortality and cannot infer causality. Second, although the subgroup analyses revealed heterogeneity in the predictive effect of GLR, the underlying pathophysiological mechanisms for the reduced or absent predictive performance in patients with high SAPSII or APSIII scores and severe liver disease require further basic and clinical research. Third, despite utilizing a dual-database, multicenter cohort design, the study is still retrospective and may be subject to unrecognized confounding factors (such as therapeutic use of insulin, types of infectious pathogens, and nutritional support interventions). Consequently, our findings demonstrate an association and should not be interpreted as causation. Furthermore, we only used a single, early, static GLR value, which does not reflect the dynamic nature of the disease process. The optimal cutoff for GLR also differed between our two cohorts, likely due to differences in baseline population risk, which limits the generalizability of a single threshold. Therefore, large-scale prospective studies with serial GLR measurements are warranted to confirm these findings and to explore the prognostic value of GLR trajectories.

In clinical practice, GLR may serve as a simple, low-cost adjunctive tool for early risk stratification in sepsis-induced ARDS patients, particularly those without severe liver dysfunction or extreme physiological derangement. It could help clinicians identify high-risk patients who may benefit from closer monitoring or intensified supportive care. Future studies should explore dynamic GLR trends and its integration into real-time clinical decision support systems.

## 5 Conclusion

This study clarifies the association between GLR and 28-day ICU mortality in patients with sepsis-induced ARDS. The findings indicate that combining GLR with APSIII provides higher prognostic accuracy compared to using APSIII alone. Given its simplicity and cost-effectiveness, GLR may serve as a useful adjunctive tool for clinicians. When interpreted alongside established severity scores, it can support the early identification of high-risk patients with sepsis-induced ARDS and contribute to a more comprehensive prognostic evaluation, thereby facilitating timely interventions and potentially improving outcomes.

## Data Availability

The original contributions presented in the study are included in the article/**Supplementary Material**. Further inquiries can be directed to the corresponding author.

## Supplementary material

Supplementary Figure 1 Heatmap of Correlation Matrices for Variables.

Supplementary Figure 2 Enternal data GLR ROC curve.

Supplementary Figure 3 ROC curves for predicting in-hospital mortality using multiple indicators **(a, b)**, and decision curve analysis **(c, d)**. ROC curves for predicting 28-day ICU mortality using Model 1 (SAPSII/APSIII) and Model 2 (SAPSII/APSIII + GLR). Model 1: SAPSII/APSIII; Model 2: SAPSII/APSIII + GLR.

Supplementary Figure 4 ROC curve for glucose alone.

Supplementary Figure 5 ROC curve for lymphocyte count alone.

Supplementary Figure 6 Distribution of GLR values in the derivation and external validation cohorts.

SUPPLEMENTARY TABLE 1 Display of missing information. BMI: Weight (kg) ÷ height (m)^2^; SBP: Systolic blood pressure; DBP: Diastolic blood pressure; MBP, Mean blood pressure; SpO2, Blood oxygen saturation; WBC, White blood cells; RDW: Red blood cell distribution width; PLT: Platelet; BUN: Blood urea nitrogen; GLR: log2[admission ICU blood glucose(mg/dl)/(18*lymphocytes (mmol/L)]; ALT: Alanine aminotransferase; AST: Aspartate aminotransferase; PTT: Partial thromboplastin time; PH: Potential of hydrogen; FiO2: Fraction of inspiration O2; PaO2: Partial pressure of oxygen in arterial blood; PaCO2: Partial pressure of carbon dioxide; SPO2: Saturation of peripheral oxygen; BE: Base excess; LDH: Lactate dehydrogenase; CRP:C-reactive protein; PPEP: Positive end-expiratory pressure; VT: Ventricular tachycardia; SOFA, Sepsis-related organ failure assessment score; SAPSII, Simplified acute physiology score II; APSIII, Acute physiology score III; Sirs: Systemic inflammatory response syndrome; AKI: Acute kidney injury; ARDS: Acute respiratory distress syndrome.

SUPPLEMENTARY TABLE 2 Display of missing information (Variables missing at 20%). LDH: Lactate dehydrogenase; CRP: C-reactive protein.

SUPPLEMENTARY TABLE 3 Variance Inflation Factor (VIF) for the stability predictor. RDW, Red blood cell distribution width; PTT, Partial thromboplastin time; SAPSII, Simplified acute physiology score II; APSIII, Acute Physiology Score III.

SUPPLEMENTARY TABLE 4 Multivariate Cox regression analysis assessed 28-day ICU mortality using external data (Xuzhou Medical University Affiliated Hospital). RDW, Red blood cell distribution width; PTT, Partial thromboplastin time; SAPSII, Simplified acute physiology score II; APSIII, Acute Physiology Score III.

## Glossary

AIDS: Acquired immunodeficiency syndrome
AKI: Acute kidney injury
AMI: Acute myocardial infarction
APS III/APSIII: Acute Physiology Score III
ARDS: Acute respiratory distress syndrome
ASCVD: Atherosclerotic cardiovascular disease
AST: Aspartate aminotransferase
ALT: Alanine aminotransferase
ALB: Albumin
APTT/PTT: Activated partial thromboplastin time
AUC: Area under the curve
BE: Base excess
BIDMC: Beth Israel Deaconess Medical Center
BMI: Body mass index
BUN: Blood urea nitrogen
CI: Confidence interval
CITI: Collaborative Institutional Training Initiative
Cox: Cox proportional hazards model
CRP: C-reactive protein
DCA: Decision curve analysis
DBP: Diastolic blood pressure
FiO₂: Fraction of inspired oxygen
GLR: Glucose-to-lymphocyte ratio
Hb: Hemoglobin
HCT: Hematocrit
HR: Heart rate/Hazard ratio
ICD-9/10: International Classification of Diseases, 9th/10th revision
ICU: Intensive care unit
IQR: Interquartile range
IRB: Institutional Review Board
KM: Kaplan–Meier
Lac: Lactate
LASSO: Least absolute shrinkage and selection operator
LDH: Lactate dehydrogenase
MAP/MBP: Mean arterial pressure
MICE: Multiple imputation by chained equations
MIMIC-IV: Medical Information Mart for Intensive Care IV
NIH: National Institutes of Health
OR: Odds ratio
PaCO₂: Partial pressure of carbon dioxide in arterial blood
PaO₂: Partial pressure of oxygen in arterial blood
PEEP: Positive end-expiratory pressure
PLT: Platelet
RCS: Restricted cubic spline
RDW: Red cell distribution width
ROC: Receiver operating characteristic
RR: Respiratory rate
SAPS II/SAPSII: Simplified Acute Physiology Score II
SBP: Systolic blood pressure
SIRS: Systemic inflammatory response syndrome
SOFA: Sequential Organ Failure Assessment
SpO₂: Peripheral oxygen saturation
SQL: Structured Query Language
STROBE: Strengthening the Reporting of Observational Studies in Epidemiology
TB: Total bilirubin
VT: Tidal volume
WBC: White blood cell
