# Supplementary figures and images for: Association of glucose–lymphocyte ratio and short-term mortality in patients with sepsis complicated by ARDS during the acute phase: a multicenter retrospective cohort study
Source: Front Cell Infect Microbiol. 2026 Mar 19;16:1771620. doi: 10.3389/fcimb.2026.1771620 (PMC13044126; doi:10.3389/fcimb.2026.1771620)

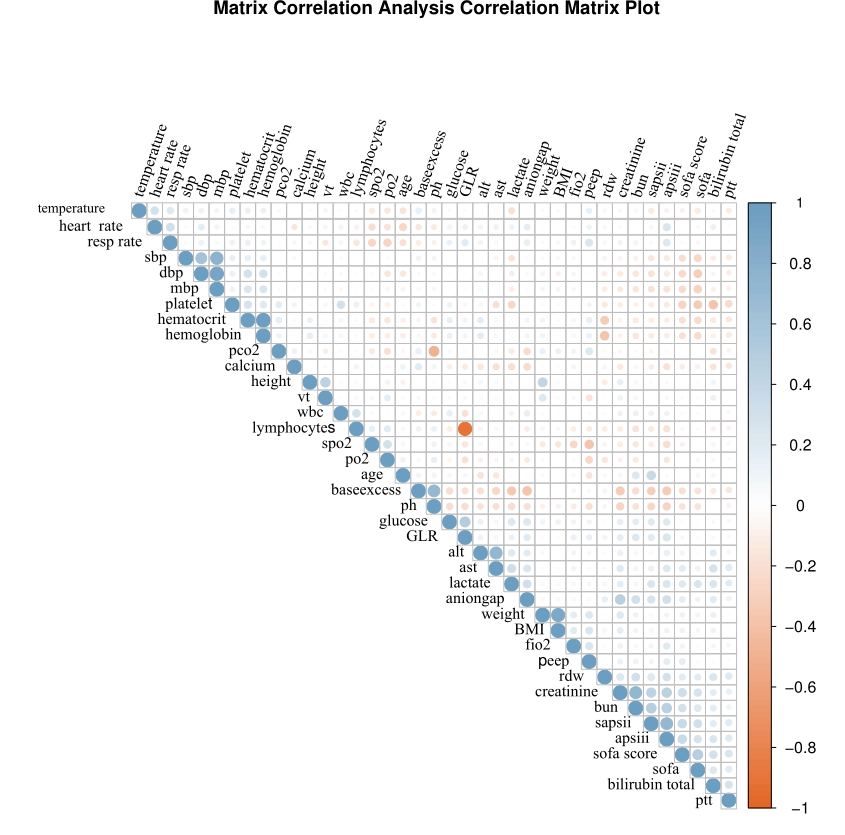

Supplement: Supplementary Figure 1 — Heatmap of Correlation Matrices for Variables. [file Image1.tif]

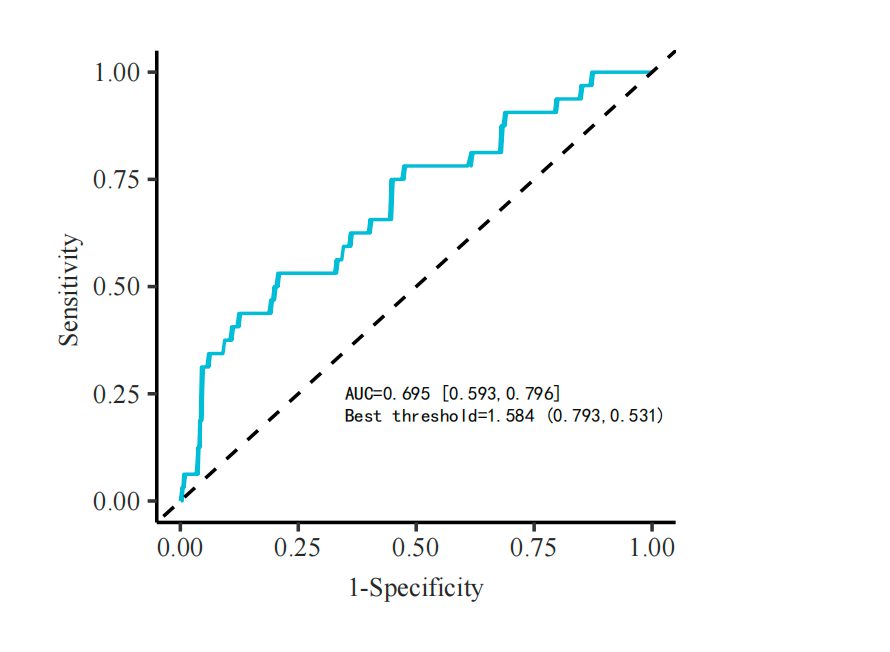

Supplement: Supplementary Figure 2 — Enternal data GLR ROC curve. [file Image2.tif]

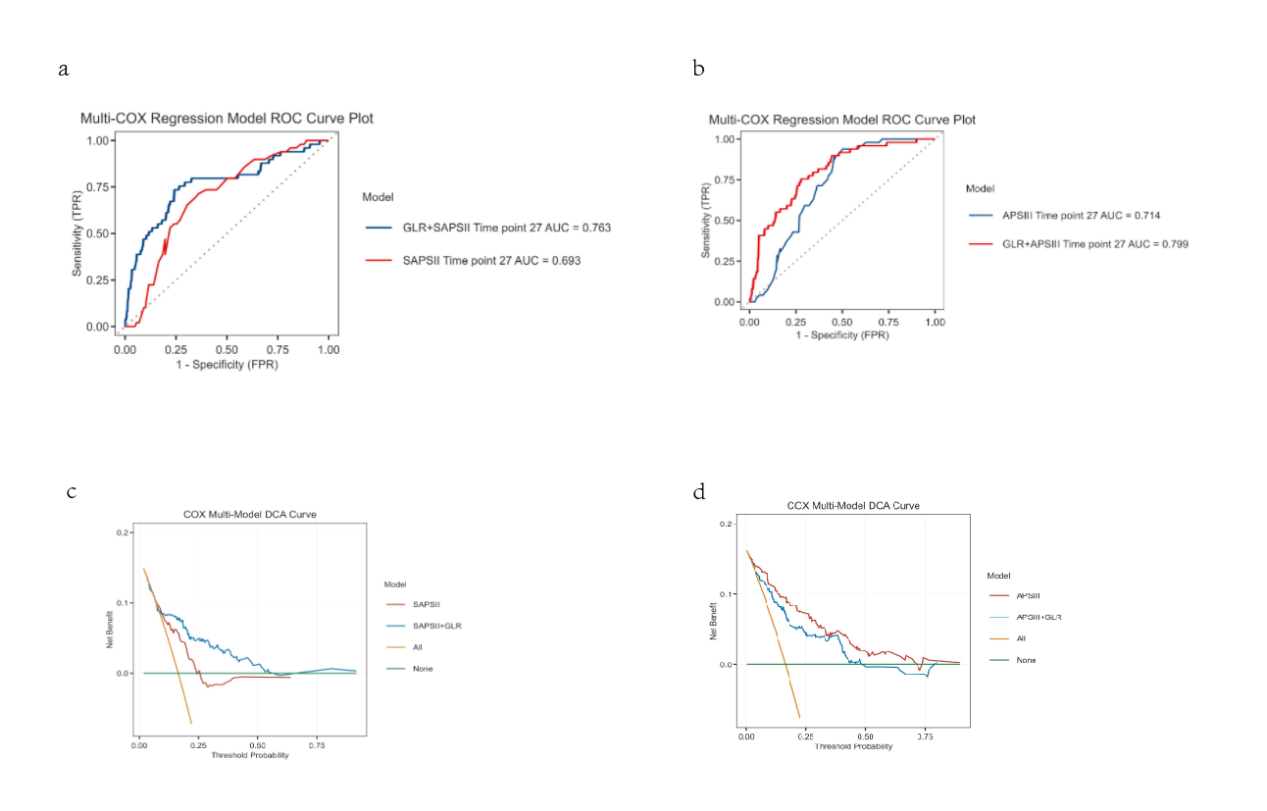

Supplement: Supplementary Figure 3 — ROC curves for predicting in-hospital mortality using multiple indicators (a, b), and decision curve analysis (c, d). ROC curves for predicting 28-day ICU mortality using Model 1 (SAPSII/APSIII) and Model 2 (SAPSII/APSIII + GLR). Model 1: SAPSII/APSIII; Model 2: SAPSII/APSIII + GLR. [file Image3.tif]

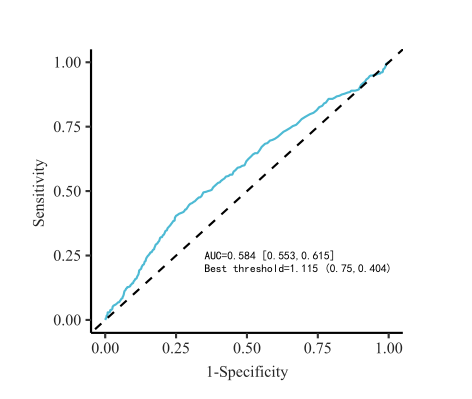

Supplement: Supplementary Figure 4 — ROC curve for glucose alone. [file Image4.tif]

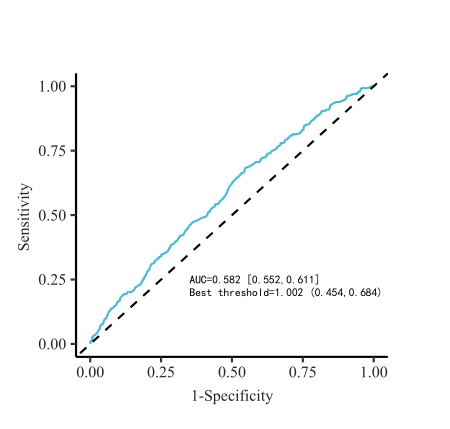

Supplement: Supplementary Figure 5 — ROC curve for lymphocyte count alone. [file Image5.tif]

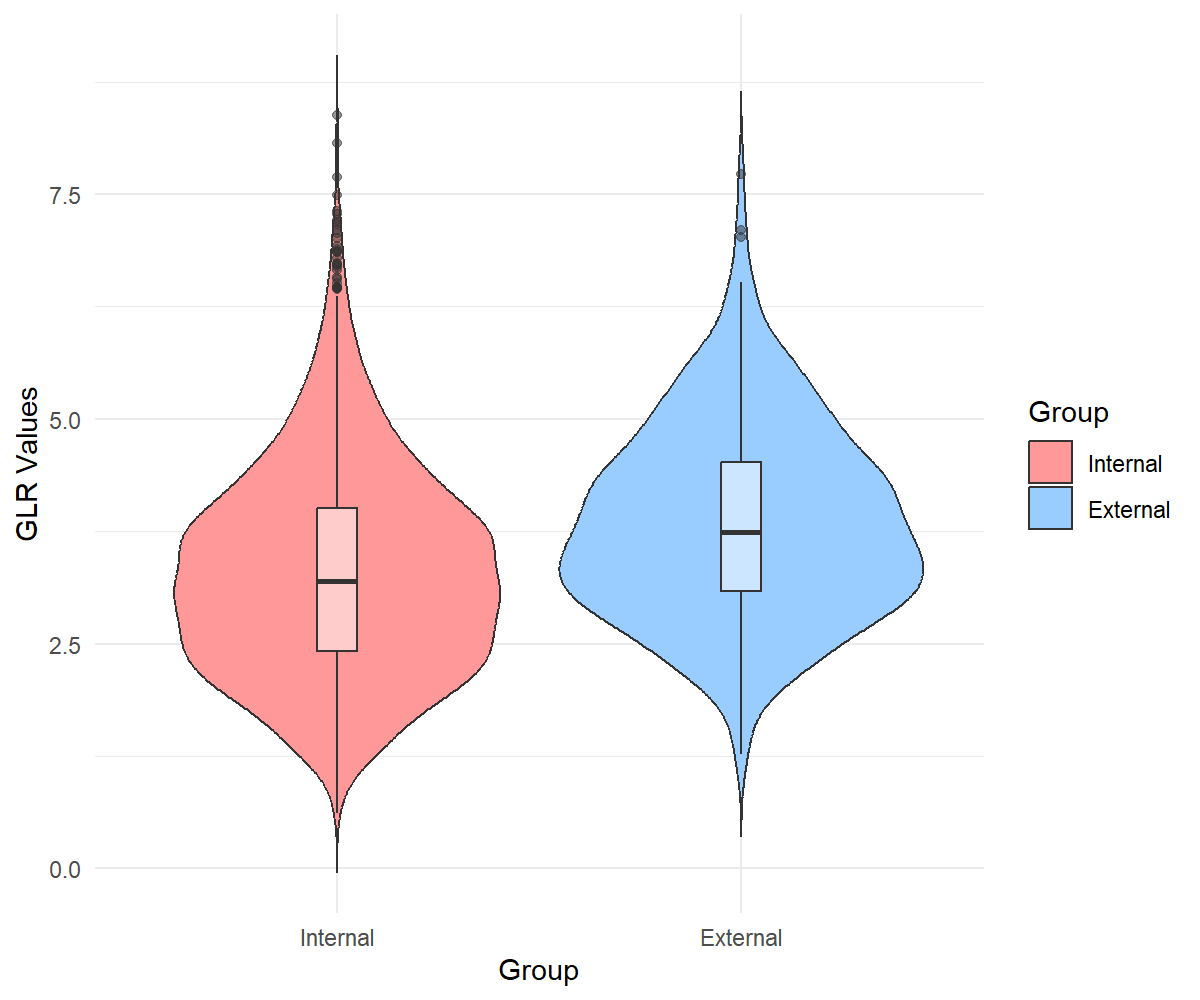

Supplement: Supplementary Figure 6 — Distribution of GLR values in the derivation and external validation cohorts. [file Image6.tiff]
