# Supplementary material for: Association of glucose–lymphocyte ratio and short-term mortality in patients with sepsis complicated by ARDS during the acute phase: a multicenter retrospective cohort study
Source: Front Cell Infect Microbiol. 2026 Mar 19;16:1771620. doi: 10.3389/fcimb.2026.1771620 (PMC13044126; doi:10.3389/fcimb.2026.1771620)
Supplement: SUPPLEMENTARY TABLE 1 — Display of missing information. BMI: Weight (kg) ÷ height (m)2; SBP: Systolic blood pressure; DBP: Diastolic blood pressure; MBP, Mean blood pressure; SpO2, Blood oxygen saturation; WBC, White blood cells; RDW: Red blood cell distribution width; PLT: Platelet; BUN: Blood urea nitrogen; GLR: log2[admission ICU blood glucose(mg/dl)/(18*lymphocytes (mmol/L)]; ALT: Alanine aminotransferase; AST: Aspartate aminotransferase; PTT: Partial thromboplastin time; PH: Potential of hydrogen; FiO2: Fraction of inspiration O2; PaO2: Partial pressure of oxygen in arterial blood; PaCO2: Partial pressure of carbon dioxide; SPO2: Saturation of peripheral oxygen; BE: Base excess; LDH: Lactate dehydrogenase; CRP:C-reactive protein; PPEP: Positive end-expiratory pressure; VT: Ventricular tachycardia; SOFA, Sepsis-related organ failure assessment score; SAPSII, Simplified acute physiology score II; APSIII, Acute physiology score III; Sirs: Systemic inflammatory response syndrome; AKI: Acute kidney injury; ARDS: Acute respiratory distress syndrome. [file Table1.docx]

**Table S1 Display of missing information.**

| **Variables** | **Proportion of missing values（%）** |
| --- | --- |
| Gender, n(%) | 0 |
| Height（cm） | 12.8 |
| Weight（kg） | 9.6 |
| **Comorbiditiesn, n(%)** |  |
| Congestive heart failure | 0 |
| Diabetes | 0 |
| Chronic pulmonary disease | 0 |
| Cerebrovascular disease | 0 |
| Myocardial infarct | 0 |
| Renal disease | 0 |
| Severe liver disease | 0 |
| AKI | 0 |
| **Vital signs** | 0 |
| Temperature (℃） | 0.3 |
| Heart rate (beats/min) | 0 |
| Resp rate (beats/min) | 0 |
| SBP (mmHg) | 0 |
| DBP (mmHg) | 0 |
| MAP (mmHg) | 0.1 |
| SpO_2_ (%) | 0 |
| **Laboratory tests** |  |
| GLR | 0 |
| WBC (K/uL) | 0 |
| Hemoglobin (g/L) | 0 |
| Thrombin （U） | 98.8 |
| Bicarbonate（mmol/L） | 92.6 |
| Hematocrit（L/L） | 0 |
| RDW (%) | 0 |
| Lymphocytes（109/L） | 0 |
| PLT (K/uL) | 0 |
| Creatinine（mg/dl） | 0 |
| BUN（mg/dl） | 0 |
| ALT（U/L） | 12.6 |
| AST（U/L） | 12.3 |
| bilirubin_indirect（μmol／L） | 79.3 |
| Bilirubin_total（μmol／L） | 12.4 |
| PTT（s） | 0.4 |
| Glucose（mg/dl） | 0 |
| PH | 0 |
| FiO_2_（%） | 0 |
| PaO_2_（mmHg） | 0 |
| PaCO_2_（mmHg） | 0 |
| Lactate（mmol/L） | 2.8 |
| Calcium（mg/dl） | 11.5 |
| Sodium（mg/dl） | 49.9 |
| Potassium（mg/dl） | 33.2 |
| BE（mmol/L） | 0 |
| LDH (U/L) | 25.5 |
| CRP（mg/L） | 87.1 |
| Albumin (g/L) | 22.8 |
| Aniongap (mEq/L) | 0 |
| **Ventilation settings** |  |
| PEEP（cmH_2_O） | 0 |
| VT（ml） | 10.8 |
| **Disease severity score** |  |
| SAPSII | 0 |
| APSIII | 0 |
| SOFA | 0 |
| ARDS | 0 |
| **Treatments, n(%)** |  |
| Vasoactive | 0 |

Abbreviations: BMI : Weight (kg) ÷ height (m)² ; SBP: Systolic blood pressure; DBP: Diastolic blood pressure; MBP, Mean blood pressure; SpO2, Blood oxygen saturation; WBC, White blood cells; RDW: Red blood cell distribution width; PLT: Platelet; BUN: Blood urea nitrogen; GLR: log_2_[admission ICU blood glucose（mg/dl）/（18*lymphocytes（mmol/L）]; ALT: Alanine aminotransferase; AST: Aspartate aminotransferase; PTT: Partial thromboplastin time; PH: Potential of hydrogen; FiO_2_: Fraction of inspiration O2; PaO2: Partial pressure of oxygen in arterial blood; PaCO2: Partial pressure of carbon dioxide; SPO2: Saturation of peripheral oxygen; BE: Base excess; LDH：Lactate dehydrogenase; CRP:C-reactive protein; PPEP: Positive end-expiratory pressure; VT: Ventricular tachycardia; SOFA, Sepsis-related organ failure assessment score; SAPSII, Simplified acute physiology score II; APSIII, Acute physiology score III; Sirs: Systemic inflammatory response syndrome; AKI: Acute kidney injury; ARDS: Acute respiratory distress syndrome.
