# Supplementary material for: Association of glucose–lymphocyte ratio and short-term mortality in patients with sepsis complicated by ARDS during the acute phase: a multicenter retrospective cohort study
Source: Front Cell Infect Microbiol. 2026 Mar 19;16:1771620. doi: 10.3389/fcimb.2026.1771620 (PMC13044126; doi:10.3389/fcimb.2026.1771620)
Supplement: SUPPLEMENTARY TABLE 2 — Display of missing information (Variables missing at 20%). LDH: Lactate dehydrogenase; CRP: C-reactive protein. [file Table2.docx]

**Table S2 Display of missing information (Variables missing at 20%).**

| Variable Names | Proportion of missing values（%） |
| --- | --- |
| Thrombin （U） | 98.8 |
| Bicarbonate（mmol/L） | 92.6 |
| bilirubin_indirect（μmol／L） | 79.3 |
| Sodium（mg/dl） | 49.9 |
| Potassium（mg/dl） | 33.2 |
| LDH (U/L) | 25.5 |
| CRP（mg/L） | 87.1 |
| Albumin (g/L) | 22.8 |

Abbreviations: LDH: Lactate dehydrogenase; CRP: C-reactive protein.
