# Supplementary material for: Association of glucose–lymphocyte ratio and short-term mortality in patients with sepsis complicated by ARDS during the acute phase: a multicenter retrospective cohort study
Source: Front Cell Infect Microbiol. 2026 Mar 19;16:1771620. doi: 10.3389/fcimb.2026.1771620 (PMC13044126; doi:10.3389/fcimb.2026.1771620)
Supplement: SUPPLEMENTARY TABLE 3 — Variance Inflation Factor (VIF) for the stability predictor. RDW, Red blood cell distribution width; PTT, Partial thromboplastin time; SAPSII, Simplified acute physiology score II; APSIII, Acute Physiology Score III. [file Table3.docx]

**Table S3 Variance Inflation Factor (VIF) for the stability predictor.**

| **Variable Names** | **VIF** |
| --- | --- |
| Age | 1.550 |
| Hematocrit | 1.262 |
| RDW | 1.513 |
| Bilirubin | 1.667 |
| PTT | 1.049 |
| Lactate | 1.598 |
| Aniongap | 1.663 |
| SapsII | 3.796 |
| ApsIII | 3.774 |
| GLR | 1.112 |
| Severe liver disease | 1.728 |

Abbreviations: RDW, Red blood cell distribution width; PTT, Partial thromboplastin time; SAPSII, Simplified acute physiology score II; APSIII, Acute Physiology Score III.
