# Supplementary material for: Association of glucose–lymphocyte ratio and short-term mortality in patients with sepsis complicated by ARDS during the acute phase: a multicenter retrospective cohort study
Source: Front Cell Infect Microbiol. 2026 Mar 19;16:1771620. doi: 10.3389/fcimb.2026.1771620 (PMC13044126; doi:10.3389/fcimb.2026.1771620)
Supplement: SUPPLEMENTARY TABLE 4 — Multivariate Cox regression analysis assessed 28-day ICU mortality using external data (Xuzhou Medical University Affiliated Hospital). RDW, Red blood cell distribution width; PTT, Partial thromboplastin time; SAPSII, Simplified acute physiology score II; APSIII, Acute Physiology Score III. [file Table4.docx]

**Table S4 Multivariate Cox regression analysis assessed 28-day ICU mortality using external data (Xuzhou Medical University Affiliated Hospital).**

| **Characteristics** | **Number (%)** | **HR (95% CI)** | ***P* value** |
| --- | --- | --- | --- |
| Age | 60.620 (13.070) | 1.009（0.983 ~ 1.036） | 0.486 |
| Hematocrit | 32.290 (7.310) | 1.027（0.986 ~ 1.070） | 0.198 |
| RDW | 34.410 (13.670) | 0.969（0.940 ~ 0.940） | 0.041 |
| Bilirubin | 29.410 (39.050) | 0.997（0.988 ~ 1.006） | 0.476 |
| PTT | 25.240 (9.900) | 1.012（0.971 ~ 1.055） | 0.567 |
| Lactate | 2.760 (2.160) | 1.071（0.942 ~ 1.217） | 0.296 |
| Aniongap | 16.870 (5.440) | 1.001（0.948 ~ 1.056） | 0.980 |
| SapsII | 37.220 (11.540) | 1.028（1.002 ~ 1.055） | 0.033 |
| ApsIII | 59.260 (21.880) | 1.022（1.006 ~ 1.038） | 0.008 |
| GLR | 3.850 (1.080) | 1.845（1.452 ~ 2.345） | ＜0.001 |
| Severe liver disease |  | 1.479（0.667 ~ 3.277） | 0.335 |
| 0 | 266 (89.300) |  |  |
| 1 | 32 (10.700) |  |  |

Abbreviations: RDW, Red blood cell distribution width; PTT, Partial thromboplastin time; SAPSII, Simplified acute physiology score II; APSIII, Acute Physiology Score III.
